# Ultra-Light Poly(N-isopropylacrylamide) Hydrogels: Light Weight Water Materials for Passive Thermal Management via Insulation and Cooling

**DOI:** 10.1007/s40820-025-02057-9

**Published:** 2026-01-28

**Authors:** Xueyan Hu, Siyuan Dou, Yiming Liu, Yaru Li, Caixia Yu, Jin Wang

**Affiliations:** 1 Key Laboratory of Multifunctional Nanomaterials and Smart Systems, Suzhou Institute of Nano-Tech and Nano-Bionics, Chinese Academy of Sciences, Suzhou, 215123 People’s Republic of China; 2 School of Nano-Tech and Nano-Bionics, University of Science and Technology of China, Hefei, 230026 People’s Republic of China

**Keywords:** Lightweight water materials, Hydrogel, Passive cooling, Thermal management, Low density

## Abstract

Water, despite its abundance, high heat capacity, and environmental benignity, has long been constrained by its intrinsic density (~ 1.0 g cm^−3^) and fluidic nature, which limit its use as a lightweight, structurally stable material above the freezing point. Reconfiguring water into an ultra-light yet solid-like form while retaining its inherent thermal and optical advantages is therefore of great significance for next-generation cooling technologies that demand low mass, portability, and sustainability. Herein, we report an ultra-light hydrogel based on poly(N-isopropylacrylamide), in which hollow foaming microspheres are incorporated to create ultra-low-density water materials. By confining water within this composite network, the hydrogel achieves a record-low density of 0.041 g cm^−3^ while maintaining a high water content of 52.7 wt%. The microspheres generate sealed air pockets that serve as highly effective thermal barriers, yielding a thermal conductivity of only 0.034–0.039 W m^−1^ K^−1^ and enabling a > 50 °C temperature differential in hot-stage tests. Furthermore, the hydrogel exhibits excellent spectral properties, with high solar reflectance (0.94) and high infrared emittance (0.84), resulting in a sub-ambient cooling of up to 10.8 °C in outdoor experiments. The synergy of ultra-low density, mechanical robustness, and multifunctional thermal regulation demonstrates a viable pathway toward practical light water materials for energy-efficient, portable, and sustainable thermal management.

**Supplementary Information:**

The online version contains supplementary material available at 10.1007/s40820-025-02057-9.

## Highlights

- The hydrogel achieves an ultra-light water-based structure (0.041 g cm^−3^, 52.7 wt% water) by incorporating hollow foaming microspheres.
- Sealed air pockets enable ultra-low thermal conductivity (0.034–0.039 W m^−1^ K^−1^) and over 50 °C thermal shielding under high-temperature conditions.
- High solar reflectance (0.94) and infrared emittance (0.84) deliver up to 10.8 °C sub-ambient outdoor cooling.

**Supplementary Information:**

The online version contains supplementary material available at 10.1007/s40820-025-02057-9.

## Graphical abstract

**Figure:**
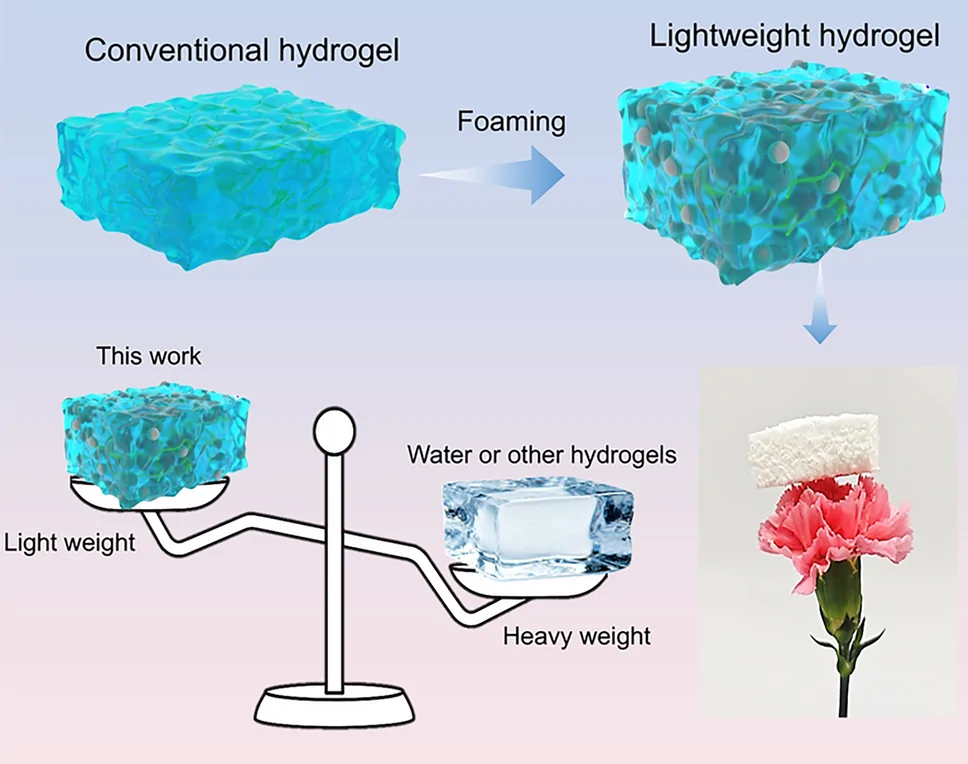

## Introduction

Water covers approximately 71% of the Earth’s surface, existing in vast quantities across oceans, lakes, rivers, glaciers, and atmospheric moisture [1–3]. Beyond being a fundamental component of the global ecosystem, water is indispensable to human civilization, supporting biological metabolism, agricultural production, industrial processing, and environmental regulation [4, 5]. Its ubiquity and benign nature make it one of the most abundant and sustainable resources available on the planet [6]. However, in its most common liquid state, water lacks a defined shape and is inherently fluid at ambient temperatures [7]. While this property allows it to adapt to diverse environments and participate in complex transport and exchange processes, it also imposes intrinsic constraints on its use as a structural or functional material [8, 9]. Encapsulation, containment, or continuous circulation systems are typically required to maintain water in place, limiting its direct applicability in load-bearing or shape-dependent applications [10].

**Fig. 1:**
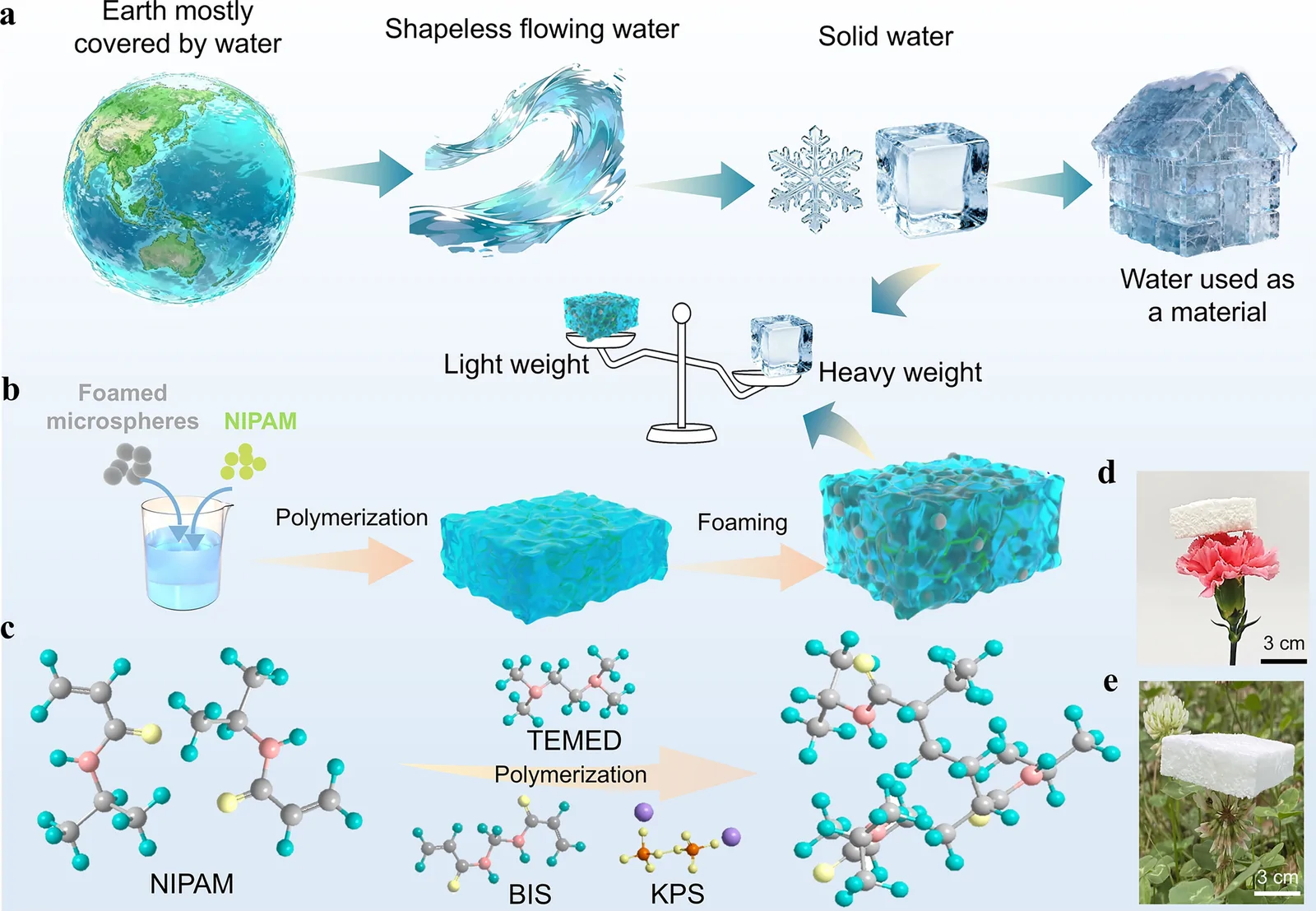
Preparation of light water materials. **a** A schematic diagram of the state of water and solid ice that can be used as building materials. **b** Synthetic steps of lightweight water materials. **c** Ball-and-stick model illustrating the polymerization and network formation of the hydrogel. **d** Lightweight water materials are placed on carnations. **e** Lightweight water materials placed on the flower

Under low-temperature conditions, water can transition into solid states such as ice or snow, acquiring defined morphology and dimensional stability [11, 12]. Frozen water has been employed in multiple regions as a temporary construction medium, from traditional igloos to large-scale ice sculptures in modern tourism (Fig. 1a). These frozen structures demonstrate that water, when immobilized, can function as an architectural or artistic material. However, the densities of water in both liquid and solid states remain approximately 1.0 g cm^−3^, which is substantially higher than that of many advanced lightweight materials [13–15]. Consequently, conventional forms of water face limitations in terms of weight burden, thermal brittleness, and environmental instability, restricting their broader utility in material science and engineering [16]. Lightweight design has therefore become a critical criterion in the development of next-generation structural and functional materials [17–20]. Reducing mass not only lowers energy consumption and transportation costs but also enhances portability, adaptability, and mechanical tunability, particularly in fields such as wearable devices, energy-efficient architecture, aerospace components, and soft robotics [21–25]. Reconfiguring water into an ultra-light yet structurally stable form while preserving its inherent advantages, such as high heat capacity, biocompatibility, and sustainability, would transform it from a passive fluid into a functional solid-state material.

Hydrogels, composed of three-dimensional polymeric networks that confine large amounts of water, provide an ideal platform for this transformation [26, 27]. Within hydrogels, water serves not merely as a filler but as the core functional component, governing thermal, optical, mechanical, and transport behaviors, while the polymeric matrix acts as a scaffold to stabilize and regulate water’s behavior [28–31]. By tailoring the network architecture, pore structure, and polymer water interactions, hydrogels can confine water into geometries and densities unattainable in its native state. Therefore, we introduce the notion of lightweight water materials (LWM), a class of ultra-low-density hydrogels in which water is retained in a structurally defined yet lightweight configuration. Unlike conventional hydrogels, which typically maintain densities near that of bulk water, LWMs are designed to minimize mass while preserving water content and functionality. This approach challenges the conventional perception of water as a dense and shapeless fluid, reimagining it as a versatile, engineered material capable of exhibiting solid-like form with minimal weight. Beyond their structural potential, LWMs are particularly relevant for thermal management applications. Water possesses a high specific heat capacity and strong infrared emissivity, which make it intrinsically suitable for heat storage and radiative dissipation. Conventional water-based systems, however, rely on liquid circulation or bulky reservoirs, which prevents their deployment in portable or passive cooling scenarios [10, 16]. When confined in a lightweight and thermally insulating matrix, water can shift from a fluid coolant to a solid-state medium that regulates temperature via radiative and evaporative pathways [30–38]. This aligns with the increasing demand for energy-efficient, lightweight, and sustainable cooling solutions, especially under conditions of global warming and urban heat island effects [39, 40]. Conventional active cooling strategies, such as air conditioning and heat exchangers, are energy-intensive and often impractical in off-grid or outdoor scenarios [41–45]. Passive cooling technologies leveraging radiative and evaporative mechanisms offer promising alternatives, yet most existing materials either suffer from excessive weight and rigidity or exhibit insufficient thermal insulation under variable environmental conditions [46–48]. Therefore, developing water-rich hydrogels that are both ultra-light and mechanically robust represents an urgent opportunity.

In this study, we report the design and fabrication of an ultra-lightweight poly(N-isopropylacrylamide) (PNIPAM)-based hydrogel incorporated with hollow foaming microspheres, achieving a record-low density of 0.041 g cm^−3^ while retaining high water content (52.7%). By systematically tuning the synthesis conditions, this LWM exhibits enhanced mechanical robustness compared to conventional PNIPAM hydrogels, along with superior thermal insulation (thermal conductivity: 0.034–0.039 W m^−1^ K^−1^) and efficient radiative cooling (solar reflectance: 0.94; infrared emittance: 0.84). These multifunctional properties result in a substantial temperature reduction of up to 10.8 °C in sub-ambient outdoor conditions and a drop exceeding 50 °C in controlled hot-stage indoor tests. Collectively, this combination of ultra-low density, mechanical integrity, and advanced thermal management demonstrates a practical realization of light water materials and highlights their potential as a versatile platform for next-generation, energy-efficient cooling technologies.

## Experimental Section

### Materials

N-isopropylacrylamide (NIPAM, 98%) was purchased from Anjanee Chemical. Prior to use, it must be purified by dissolving it in n-hexane, followed by cooling and recrystallization to obtain white crystals. Foaming microspheres were purchased from Dongjin Fine Chemicals Co., Ltd., in South Korea. N, N'-Methylenebisacrylamide (BIS, > 98%) was purchased from Shanghai Tixi'ai Chemical Industry Development Co., Ltd. Potassium persulfate (KPS, ≥ 99.5%) was purchased from Sinopharm Chemical Reagents Co., Ltd. Tetramethylenediamine (TEMED, 99%) was purchased from Shanghai Aladdin Reagent Co., Ltd. All the other reagents were used as received.

### Preparation of the PNIPAM-Based LWM

At room temperature, dissolve 2 g of NIPAM and 2, 4, 6, or 8 g of foaming microspheres in deionized water to form a uniform hydrogel precursor solution. Subsequently, the initiator (0.1 g KPS), crosslinking agent (0.15 g BIS), and accelerator (0.01 g TEMED) are each dissolved in the solution until a homogeneous solution is formed, followed by polymerization at 80, 90, or 100 °C in an oven for 1 h.

### Characterization

The structural and physical properties of the synthesized LWMs were systematically characterized as follows: Fourier transform infrared spectra of the samples were collected using a Nicolet 6700 Fourier transform infrared spectrometer in the 4000–500 cm^−1^ range. At room temperature, the thermal conductivity of the hydrogel was measured using a hot-wire thermal conductivity meter (TC3000E). Two tests were conducted with a 5-min interval between them, and the average of three measurements was taken as the final result. Thermal stability and content measurements were taken using a thermogravimetric analyzer (Netzsch) under a nitrogen flow at a heating rate of 5 °C min^−1^. Mechanical property testing was conducted using an electronic universal testing machine (Instron 3365) in compression mode, with a gauge length of 10 mm and a loading rate of 0.5 mm min^−1^. The surface morphology of the PNIPAM-microsphere hydrogel was observed using a scanning electron microscope (FEI-Quanta FEG 250) at an acceleration voltage of 20 kV. Since the samples are insulating, gold was sputter-deposited onto the samples prior to scanning electron microscopy (SEM) testing, with a 30 mA gold sputtering treatment for 180 s. The contact angle of the PNIPAM-microsphere hydrogel at different temperatures was measured using a video optical contact angle measuring instrument (OCA 15EC). The surface roughness of the hydrogel was observed using a three-dimensional confocal microscope (VK-X250K). Thermogravimetric analysis was performed using a TG 209 F1 thermogravimetric analyzer at temperatures ranging from 30 to 800 °C, with a heating rate of 10 °C min^−1^ under a nitrogen gas flow. The sample temperature was measured using a thermocouple connected to a temperature data acquisition system (JK 808), and a temperature tracking curve was obtained by recording continuous data. Thermal images were captured by an infrared camera (RT630). Density is calculated based on the ratio of mass to volume (where each sample is measured three times and the average is taken).

## Results and Discussion

### Synthesis of Ultra-Light Hydrogels

As shown in Fig. 1b, to obtain a hydrogel with both low density and high water retention, a composite structure was constructed by embedding expandable microspheres into a PNIPAM matrix. The hydrogels were synthesized via free-radical polymerization using NIPAM as the monomer, KPS as the initiator, BIS as the crosslinker, and TEMED as the accelerator (Fig. 1c). Before polymerization, hollow foaming microspheres were premixed into the monomer solution. Upon heating during gelation, the microspheres expanded and became immobilized within the forming three-dimensional network, yielding a lightweight PNIPAM hydrogel. The ultra-low density of the hydrogels is further evidenced by their ability to remain stably supported on carnations and flowers (Fig. 1d, e), indicating a mass sufficiently low to avoid perceptible mechanical loading on fragile substrates.

### Characterization of Ultra-Light Hydrogels

**Fig. 2:**
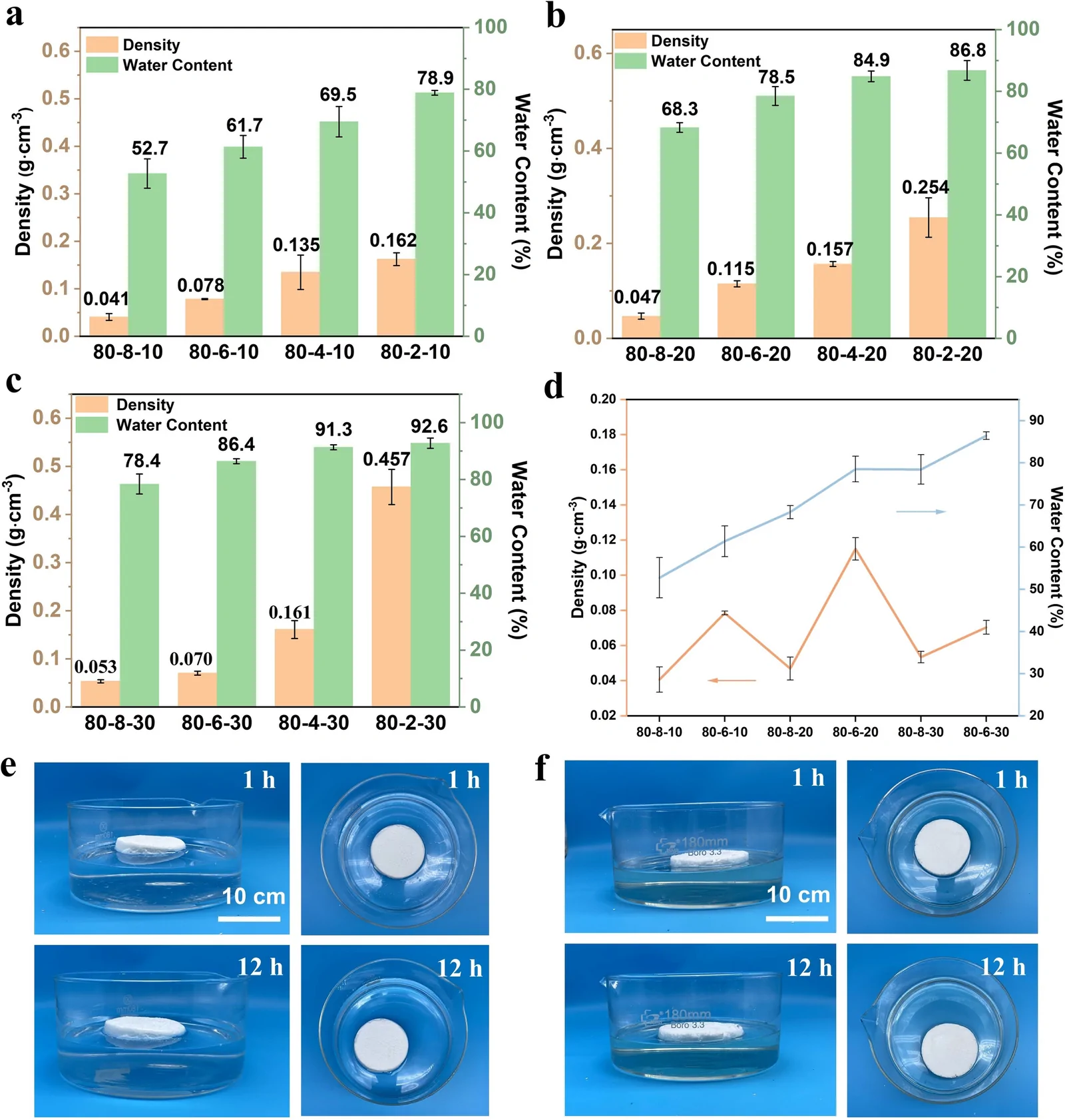
Density and water content of the LWMs. **a–c** Bar charts showing the density and water content of the ultra-light hydrogels with crosslinking temperatures of 80 °C and deionized water additions of 10 mL, 20 mL, and 30 mL, respectively. (The error bars represent the standard deviation obtained from three measurements.) (The error bars represent the standard deviation obtained from three measurements.) **d** Summary of density and water content for six hydrogel samples. (The error bars represent the standard deviation obtained from three measurements.) **e** Photograph images of the LWM80-8-10 hydrogel after soaking in deionized water for 1 h and 12 h. **f** Photograph images of the LWM80-8-10 hydrogel after soaking in hexane for 1 h and 12 h

For clarity of identification, the PNIPAM ultra-light hydrogels synthesized in this study were labeled as LWMx-y-z according to the reaction temperature (x), foaming microsphere content (y), and deionized water content (z). Detailed synthesis parameters corresponding to each sample are provided in the experimental section under hydrogel preparation. A controlled variable approach was adopted to evaluate the influence of the NIPAM-to-foaming microsphere mass ratio, deionized water content, and crosslinking temperature on the density and water content of the ultra-light hydrogels. The density and water content of the LWMs exhibited strong correlations with both the synthesis temperature and the composition of foaming microspheres and deionized water. The corresponding results are shown in Figs. 2a–f and S1. Each bar chart compares the density and water content of hydrogels synthesized under the same crosslinking temperature and deionized water addition while varying only the NIPAM-to-foaming microsphere mass ratio. Figure 2a–c presents results obtained at 80 °C with 10, 20, and 30 mL of deionized water, respectively, while Fig. 2d–f shows corresponding data for 90 °C, and Fig. S1 summarizes results at 100 °C.

As shown in Fig. 2a, samples prepared at 80 °C exhibited densities ranging from 0.041 to 0.162 g cm^−3^, demonstrating that the densities could be widely tuned through formulation control. Specifically, the sample LWM80-8-10 exhibited the lowest density of 0.041 g cm^−3^ with a water content of 52.7%, owing to the combined effects of high microsphere loading and moderate water addition, which enabled efficient foaming and formation of highly porous cellular structures. As the microsphere content decreased, the internal voids became less abundant, and the density gradually increased to 0.078 g cm^−3^ for LWM80-6-10, 0.135 g cm^−3^ for LWM80-4-10, and finally 0.162 g cm^−3^ for LWM80-2-10, highlighting the dominant role of foaming microspheres in regulating pore volume and matrix compactness. The influence of water content was also pronounced. At a fixed microsphere ratio of 8 g, increasing the deionized water content from 10 to 20 and 30 mL (corresponding to water fractions of 52.7%, 68.3%, and 78.4%, respectively) led to a moderate rise in density from 0.041 g cm^−3^ (LWM80-8-10) to 0.047 g cm^−3^ (LWM80-8-20) and 0.053 g cm^−3^ (LWM80-8-30) (Fig. 2a–c). Although higher water content typically promotes network swelling, the concurrent increase in solid-phase dispersion and secondary crosslinking can enhance matrix stability, preventing excessive collapse during drying. Interestingly, even at an extremely high water content of 91.3%, the sample LWM80-4-30 maintained a relatively high density of 0.161 g cm^−3^, suggesting that its denser polymer framework, resulting from reduced microsphere loading and intensified crosslinking, effectively expands and preserves structural integrity under high hydration conditions.

Temperature further influenced the compactness of the hydrogel network. Under identical compositions, elevating the synthesis temperature from 80 to 100 °C led to a significant density increase, from 0.041 g cm^−3^ (LWM80-8-10) to 0.106 g cm^−3^ (LWM100-8-10) (Fig. S1). This phenomenon is attributed to accelerated polymer crosslinking and partial collapse of the foamed structure at higher temperatures, yielding a denser and more consolidated matrix. Consequently, hydrogels synthesized at 80 °C consistently exhibited the lowest densities, as the moderate reaction temperature allowed full microsphere expansion and efficient foaming, reducing the overall solid mass fraction. Based on these findings, representative samples with relatively high microsphere contents, namely LWM80-8-30, 80-8-20, 80-8-10, 80-6-30, 80-6-20, and 80-6-10, were selected for further comparison (Fig. 2g). When the NIPAM-to-microsphere ratio was constant, the density exhibited a gradual increase with increasing water content. For instance, the densities of LWM80-8-10, 80-8-20, and 80-8-30 are 0.041, 0.047, and 0.053 g cm^−3^, respectively. Conversely, at a fixed water addition, samples with a NIPAM-to-microsphere mass ratio of 1:4 (0.041 g cm^−3^) consistently showed lower densities than those with a ratio of 1:3 (0.078 g cm^−3^), confirming the critical role of microsphere-induced foaming efficiency in determining the material’s lightweight characteristics.

Across all tested formulations, the LWM series exhibited a wide range of tunability in density, spanning from 0.041 to 0.532 g cm^−3^, while maintaining variable water contents, from moderate (52.7%) to extremely high (92.6%). The buoyancy of the ultra-light hydrogels further verified their low densities; for instance, sample LWM80-8-10 remained afloat on both deionized water and n-hexane (0.659 g cm^−3^) for over 12 h (Fig. 2h, i), confirming that its bulk density was lower than both polar and nonpolar liquids. These results collectively demonstrate that effective foaming, rather than dehydration, is the primary mechanism of mass reduction in the PNIPAM-foaming microsphere system. Such adjustable density-hydration coupling reveals the strong design flexibility of the foamed hydrogel network, enabling precise control of structural lightness and mechanical stability for advanced thermal management applications.

**Fig. 3:**
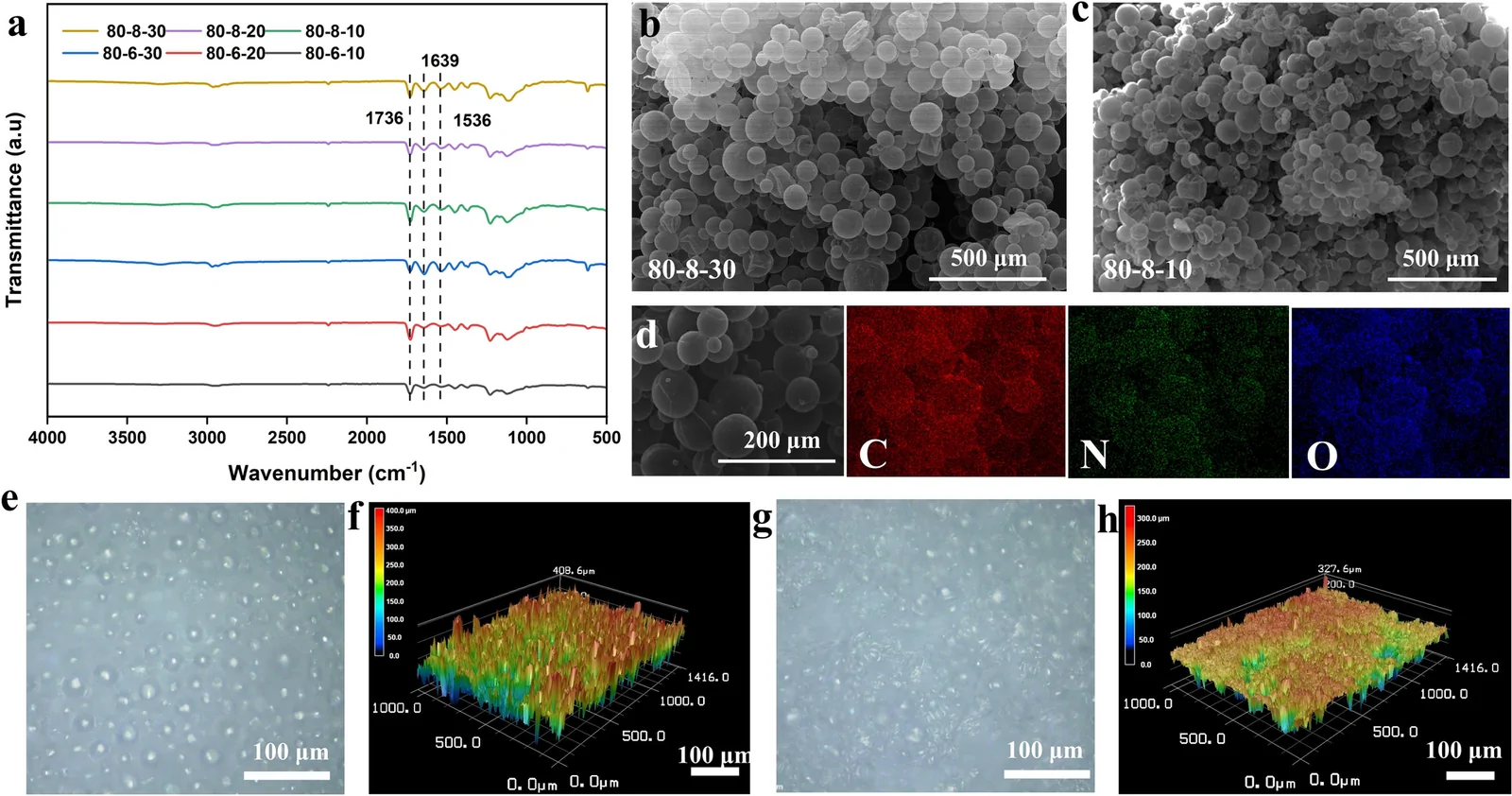
Basic characterization of the LWMs. **a** FT-IR spectroscopy of LWMs. **b** SEM image of the LWM80-8-30 dry gel. **c** SEM image of the LWM80-8-10 dry gel. **d** EDS mapping image of the LWM80-8-30 dry gel. **e** LWM80-8-30 microscopic image of the hydrogel. **f** Surface roughness of LWM80-8-30 hydrogel. **g** Microscopic image of LWM80-8-10 hydrogel. **h** LWM80-8-10 surface roughness of the hydrogel

Fourier transform infrared (FT-IR) spectroscopy confirmed the successful formation of the PNIPAM-based LWM (Fig. 3a). The pure PNIPAM hydrogel exhibits characteristic absorption bands at 1639 and 1536 cm^−1^, corresponding to the C = O stretching vibration of the amide group and the N–H bending vibration, respectively, indicating successful polymerization of NIPAM [49–51]. In contrast, a characteristic absorption band at around 1736 cm⁻^1^ (Figs. 3a and S2) attributed to the ester carbonyl (C = O) stretching vibration typical of acrylic resin materials (foaming microspheres shell) [52]. All LWM hydrogels (LWM 80-8-30, LWM 80-8-20, LWM 80-8-10, LWM 80-6-30, LWM 80-6-20, and LWM 80-6-10) display both sets of characteristic peaks, confirming that the PNIPAM polymer matrix and foaming microspheres coexist within the hybrid network. The morphology and foaming behavior of the foaming microspheres were examined by SEM. Figure S3 compares the foaming microspheres before and after thermal expansion, along with the corresponding pore size distributions. A distinct spherical structure is observed in both cases; however, after foaming, the average particle diameter increases from 18.69 to 86.08 μm, corresponding to a nearly 100-fold volumetric expansion. This substantial increase in particle volume provides an intuitive explanation for the significant reduction in the hydrogel density. Elemental mapping (Fig. S4) further confirms that C, H, and O are uniformly distributed throughout the foaming microspheres, suggesting compositional homogeneity.

Representative SEM images of the freeze-dried composite hydrogels LWM80-8-30 and LWM80-8-10 (Fig. 3b, c) show well-dispersed spherical domains embedded within the polymeric matrix. Energy-dispersive spectroscopy (EDS) mapping (Fig. 3d) again verifies the uniform distribution of C, H, and O elements. The corresponding pore size distributions (Fig. S5) show relatively narrow particle size distributions. Further statistical analysis of multiple samples (LWM80-8-30, LWM80-8-20, LWM80-8-10, LWM80-6-30, LWM80-6-20, and LWM80-6-10; Fig. S6) reveals average foaming microsphere diameters ranging from 80.77 to 93.89 μm, corresponding to 80–125-fold volumetric expansion compared to unreacted particles. These observations demonstrate that efficient foaming microsphere expansion plays a dominant role in lowering the density of the LWM hydrogel.

Three-dimensional optical profilometry was employed to characterize the surface topography of the LWM hydrogels. As shown in Figs. 3e–h and S7, the hydrogel surface exhibits pronounced roughness, with average roughness values in the range of 300–400 μm. This high degree of surface irregularity is primarily attributed to the rapid vaporization of low-boiling-point solvents within the foaming microsphere cores during polymerization, which drives the foaming microsphere expansion from approximately 18 to 80–90 μm in diameter. The subsequent mechanical compression between neighboring expanded microspheres further amplifies the undulating morphology. In addition, post-processing steps such as mechanical handling, friction, or cutting may contribute to secondary roughening of the exposed surface. In addition, the incorporation of microspheres effectively reinforces the network, allowing the hydrogel to maintain its structural stability under thermal stimuli. After three thermal cycles (30 min at ambient temperature and 30 min on an 80 °C hot plate, with each cycle lasting 1 h), the LWM retains its overall structural integrity despite the loss of water, as shown in Fig. S8.

**Fig. 4:**
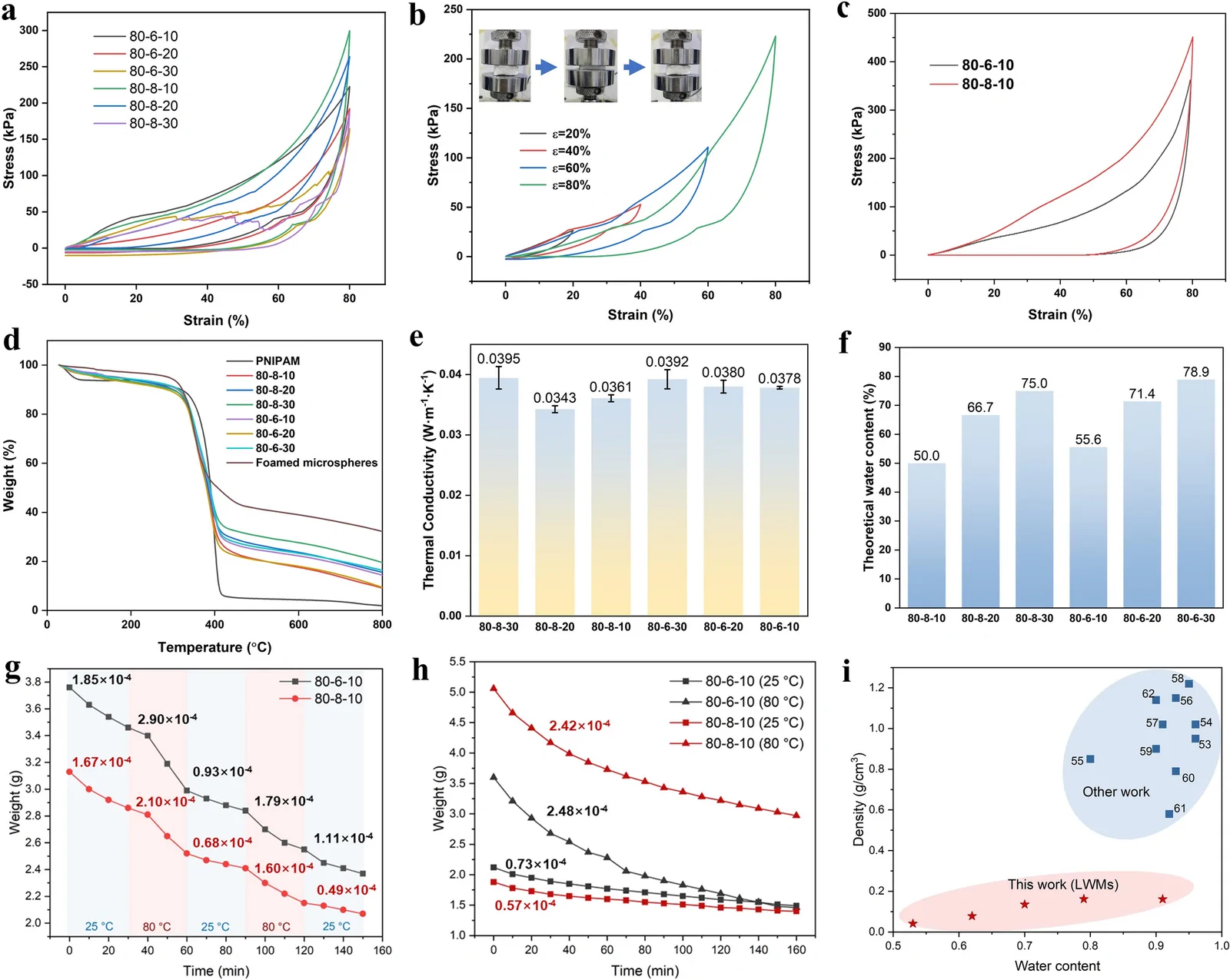
Basic characterization of LWM80-8-30, LWM80-8-20, LWM80-8-10, LWM80-6-30, LWM80-6-20, and LWM80-6-10. **a** Stress–strain curves for a single loading–unloading cycle of different samples. **b** Stress–strain curve of the LWM80-6-10 hydrogel during a single loading–unloading cycle. **c** The LWM compression stress–strain diagram after three thermal cycles.** d** TGA curves of PNIPAM dry gel and the LWM. **e** Thermal conductivity of different samples is indicated.** f** Theoretical water content of different samples. **g** LWMs mass change data and evaporation rate during the thermal cycling process.** h** LWMs mass change data and evaporation rate at different temperatures**. i** Comparison of density and water content with other hydrogels [53–62]

Beyond structural characterization, mechanical testing further confirmed that the incorporation of foaming microspheres not only reduces density but also imparts notable elasticity and recoverability to the hydrogel network. The compressive performance of the hydrogels was assessed through uniaxial loading–unloading experiments. Figure 4a presents the stress–strain curves of representative samples. All LWM hydrogels showed measurable compressive resilience. During loading, deformation is first accommodated by the collapse and distortion of internal pores within the closed-cell structure, which effectively dissipates external energy. Once the load is removed, the structure gradually rebounds, indicating recoverable deformation rather than permanent plastic collapse. However, samples LWM 80-6-30 and LWM80-8-30 exhibited structural failure at higher strain levels, likely due to excessive water content weakening interfacial adhesion between foaming microspheres and the surrounding polymer network. This observation suggests an inverse correlation between water content and compressive robustness, in which higher hydration compromises structural cohesion.

As shown in Figs. 4a and S8, increasing the microsphere content enhances the mechanical properties under the same water content. For example, the maximum stress increases from 213 kPa for the LWM80-6-10 sample to 300 kPa for the LWM80-8-10 sample. In contrast, pure PNIPAM hydrogel exhibits only 3 kPa at 60% strain (Fig. S9), whereas the LWMs reach 164–300 kPa at 80% strain, representing an improvement of more than two orders of magnitude in load-bearing capability. In addition, after 100 compression cycles, the maximum stress of the LWMs decreases by only 25.8 kPa. Figure 4b further shows the stress–strain response of sample LWM80-6-10 at different strain levels (20%, 40%, 60%, and 80%). Stress increases progressively with strain, and the increasingly steep slope reflects strain-stiffening behavior typical of elastomeric cellular materials. The inset schematics illustrate the deformation–recovery process at 80% strain, confirming that the material retains high elasticity even under substantial compressive loads, with recovery ratios approaching 80%. After three thermal cycles (30 min at ambient temperature and 30 min on an 80 °C hot plate; one cycle lasts 1 h), the dried LWMs become denser due to water loss, leading to increased maximum compressive stress, rising to 360 and 450 kPa (Fig. 4c). The enhanced resilience is primarily attributed to the gas-filled microspheres embedded within the polymeric hydrogel matrix. During compression, the encapsulated gas is compressed and temporarily stores energy; once the external force is released, rapid gas expansion promotes structural rebound. Meanwhile, the flexible polymer shell surrounding each foaming microsphere prevents rupture, allowing large reversible deformations. This synergistic mechanism enables both energy absorption and rapid recovery, contributing to the material’s durability under cyclic loading. Minor inflection points are observed in certain loading–unloading curves. This irregularity may arise from a dynamic interplay between cavity exposure and matrix compaction during deformation. As compression proceeds, internal voids gradually collapse or fill, leading to a transient equilibrium state in which stress fluctuations diminish.

The thermal stability of the LWM dried gel was investigated through thermogravimetric analysis (TGA) over a temperature range of 30–800 °C. As shown in Fig. 4d, the material remains structurally stable below 300 °C, with noticeable weight loss occurring only beyond this point. This suggests that the PNIPAM dried gel possesses sufficient thermal stability for practical applications involving moderate heat exposure, such as thermal insulation or protective packaging. In addition to thermal behavior in the solid state, the surface wettability of the hydrogel was evaluated via static contact angle measurements (Fig. S10). Although PNIPAM inherently contains hydrophobic methyl (–CH_3_) groups, which typically result in limited water affinity, the introduction of foamed microspheres dramatically alters the surface characteristics. The C=O groups present on the microsphere surface engage in strong hydrogen bonding with water molecules, significantly reducing solid–liquid interfacial tension. Furthermore, the interconnected porous architecture of the microspheres induces capillary-driven liquid infiltration. Combined with the hydrogel’s high intrinsic water content (> 90%), these effects enable rapid spreading of water droplets, causing the apparent contact angle to quickly approach nearly zero and exhibiting a superhydrophilic behavior.

To further explore its functional potential in thermal management, the thermal conductivity of the hydrated hydrogel was measured (Fig. 4e). With thermal conductivity values of 0.034–0.039 W m^−1^ K^−1^, the hydrogel can be categorized as a low-conductivity material suitable for insulation applications. This performance can be attributed to the large number of closed-cell structures formed by the foamed microspheres. These sealed cavities inhibit gas convection and minimize heat transfer through the solid framework. Additionally, the presence of water within the hydrogel introduces an evaporative cooling mechanism: During heating, water gradually vaporizes and absorbs latent heat, which further suppresses surface temperature rise. The synergistic effect of closed-cell insulation and phase change cooling endows the hydrogel with excellent thermal shielding capability. The theoretical water contents of the six representative samples were calculated to be 50%, 66.7%, 75%, 55.6%, 71.4%, and 78.9%, respectively, as shown in Fig. 4f. These values establish a quantitative foundation for evaluating the contribution of internal moisture to the overall thermal dissipation performance of LWMs. To further elucidate the role of retained water in governing thermal regulation, we systematically investigated the evaporation behavior of the LWMs under controlled thermal cycling (Fig. 4g, h). The LWM80-6-10 and LWM80-8-10 samples were subjected to alternating intervals of 30 min at ambient temperature and 30 min on an 80 °C hot plate. During the initial ambient stage, the evaporation rates were relatively high, measured at 1.85 × 10^–4^ and 1.67 × 10^–4^ kg m^−2^ s^−1^, respectively. Upon heating to 80 °C, the rates increased markedly to 2.9 × 10^–4^ and 2.1 × 10^–4^ kg m^−2^ s^−1^. Following two complete cycles, the evaporation rates progressively declined as the available water content decreased. A comparable trend was observed under isothermal conditions (25 and 80 °C), as shown in Fig. 4h. Across all temperature settings and under identical water content, the LWM80-8-10 hydrogel, which contains a higher fraction of microspheres, consistently exhibited a lower evaporation rate than the LWM80-6-10 counterpart. This behavior unequivocally indicates that the incorporation of microspheres suppresses water loss by hindering moisture transport within the network, thereby facilitating more sustained water retention and enhanced thermal management capability.

In addition to the mechanical and thermal conductivity for heat insulation analyses discussed above, it is worth revisiting the density characteristics initially introduced in Fig. 2. As can be seen from Fig. 4i, a comparative analysis of density and water content across various reported hydrogels reveals a clear distinction between this work and previous studies [53–62]. Most conventional hydrogels exhibit water contents exceeding 80%, yet their densities remain relatively high, typically ranging from 0.7 to 1.3 g cm^−3^. Such densities substantially limit their applicability in lightweight or floating systems. By contrast, the hydrogels developed in this work achieve a much broader range of hydration levels (50–95%), while simultaneously maintaining ultra-low density. The lowest measured density reaches 0.041 g cm^−3^, an order of magnitude lower than that of many reported hydrogels. Even at a high water content of 91%, the density remains as low as 0.161 g cm^−3^, which is still substantially below the values commonly observed in conventional hydrogel systems. This unique combination of high water content and ultra-light structure highlights the distinct advantage of the LWM.

### Analysis of the Thermal Insulation Properties of the Ultra-Light Hydrogels

**Fig. 5:**
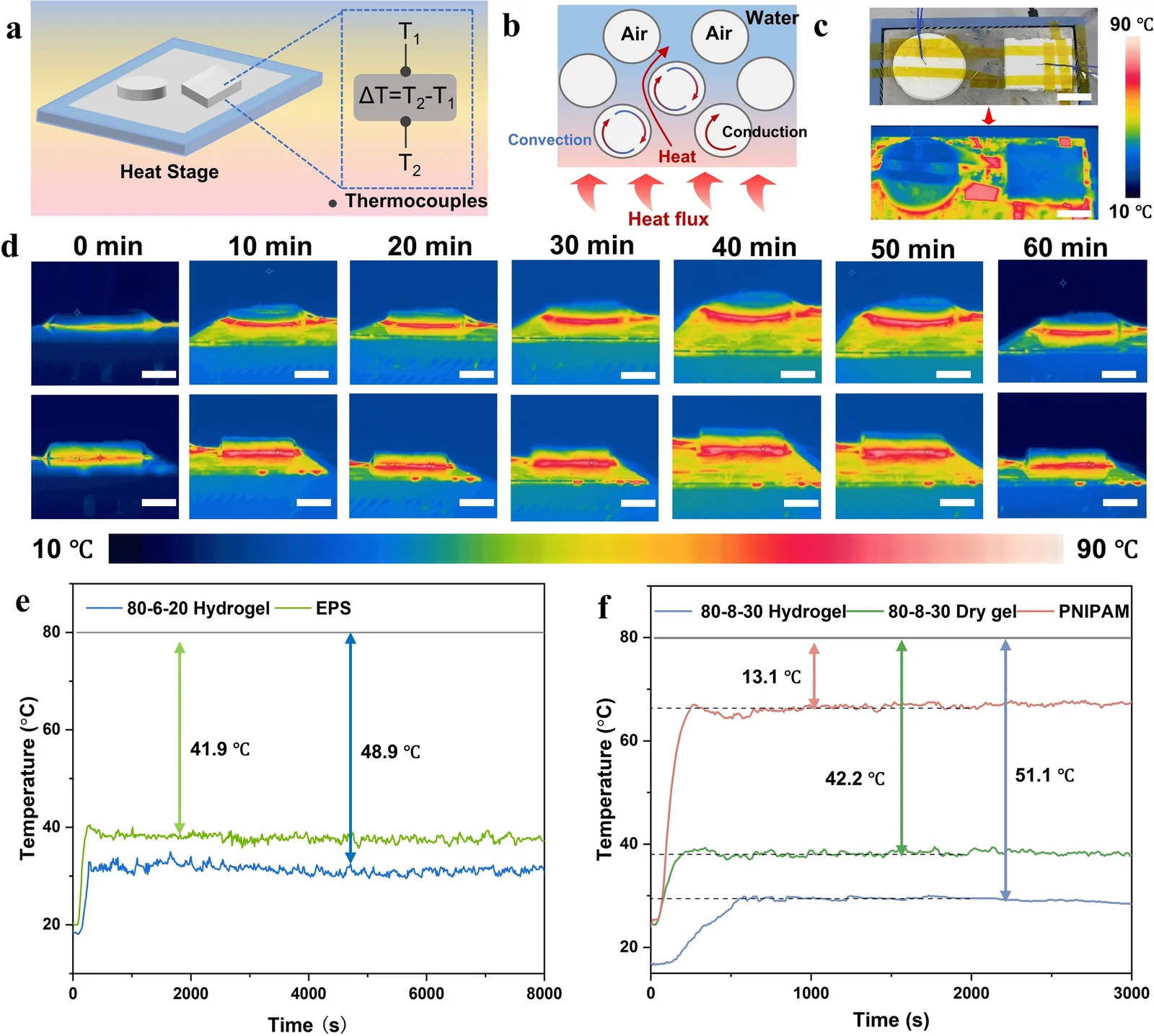
Thermal insulation performance analysis of the LWMs. **a** Schematic diagram and apparatus diagram of the hot plate experiment. **b** Schematic diagram of thermal insulation mechanism. **c** Apparatus diagram of the hot plate experiment for LWMs and expandable polystyrene, and infrared photographs of the sample surface were taken during the hot plate experiment (the set temperature of the hot stage is 80 °C) (scale bar:2 cm). **d** Infrared photographs of the material's side at different time intervals (the set temperature of the hot stage is 80 °C) (scale bar: 2 cm). **e** Temperature–time change curves for LWMs and expandable polystyrene hot plate experiments. **f** Temperature–time change curves for LWMs and pure PNIPAM hot plate experiments

To quantitatively evaluate the thermal insulation performance of the ultra-light hydrogels under well-controlled indoor conditions, a standardized hot plate experiment was conducted using two materials of identical thickness: the LWM hydrogel and a widely used commercial insulation material, expandable polystyrene (EPS). Figure 5a illustrates the schematic testing configuration and experimental setup. During testing, each sample was placed on a hot stage maintained at a fixed temperature of 80 °C. A thermocouple was firmly attached to the top surface of the sample to record real-time temperature evolution, allowing accurate tracking of heat transfer through the material. The test principle was to monitor the upper surface temperature until thermal equilibrium was reached and then calculate the temperature difference relative to the hot plate as a quantitative indicator of insulation effectiveness. The incorporation of hollow microspheres plays a pivotal role in reducing the thermal conductivity of the LWMs. Because the microspheres are internally filled entirely with air, their introduction increases the volume fraction of thermally insulating voids within the polymer water network. As illustrated by the thermal insulation mechanism diagram in Fig. 5b, this dispersed air phase disrupts continuous heat conduction pathways and markedly suppresses phonon transport through the material. In addition, the microspheres act as scattering centers that further impede thermal energy transfer by interrupting direct contact between water domains and the polymer matrix [63]. Consequently, the presence of air-filled microspheres significantly lowers the overall thermal conductivity of the LWMs, leading to superior insulation performance compared with conventional hydrogels.

Representative infrared thermographs captured during the experiment are shown in Fig. 5c. Even after prolonged exposure to the heat source, the surface regions of both samples remained in the blue temperature range, significantly lower than the preset plate temperature, confirming that both materials provided thermal resistance. Infrared images were also recorded periodically from the lateral direction to directly visualize heat propagation and compare the rate of thermal penetration between the two materials. As shown in Fig. 5d, after 10 min of heating, the upper surface of the LWM hydrogel remained distinctly cooler than that of the EPS sample, indicating a slower heat transfer rate. The temperature–time curves presented in Fig. 5e further validate this observation. At equilibrium, EPS exhibited a temperature difference of 41.87 °C with respect to the hot stage, whereas the LWM hydrogel reached a substantially higher-temperature difference of 48.85 °C, clearly demonstrating its superior insulation capability.

This performance enhancement originates from two synergistic mechanisms. First, the three-dimensional closed-cell architecture formed by the foamed microspheres significantly restricts internal air movement, thereby suppressing convective heat transfer. Second, the presence of retained water introduces an additional evaporative cooling pathway, which dissipates heat through phase change during heating, further lowering the surface temperature. To determine how compositional parameters affect thermal insulation, six LWM hydrogel formulations with varying water content and foaming microsphere loadings were subjected to identical hot plate tests. Since all test hydrogels were synthesized at 80 °C, the test temperature was likewise maintained at 80 °C to ensure consistency with the fabrication conditions and avoid any thermal interference. As shown in Fig. S11, all samples exhibited strong insulation performance, maintaining equilibrium temperature differences between 47.6 and 51.4 °C. Notably, a clear positive correlation was observed between water content and insulation efficiency, suggesting that moisture-assisted evaporative cooling is a dominant contributor to temperature regulation in these materials.

To further isolate the role of water and validate the structural contribution of the microspheres, comparative thermal stage experiments were carried out on pure PNIPAM hydrogel and on fully dried versions of the ultra-light hydrogels. As shown in Fig. 5f, pure PNIPAM hydrogel displayed a continuous surface temperature rise under heating and only reached equilibrium at 66.9 °C. Moreover, visual inspection (Fig. S12) revealed severe dehydration-induced shrinkage, indicating insufficient thermal stability. The corresponding temperature difference relative to the hot plate was merely 13.1 °C, confirming poor intrinsic insulation capacity. By contrast, the LWM80-8-30 hydrogel retained a temperature difference of 51.1 °C under the same conditions, while its dried counterpart still maintained 42.2 °C. Figure S13 further presents the thermal response curves of the dried gels derived from all six hydrogel formulations. Despite the absence of water, these materials retained considerable insulation capability, exhibiting temperature differences of 38.5–42.2 °C at 80 °C. This confirms that the macrosize porous network alone provides substantial thermal resistance by impeding convective and conductive transfer. However, in the absence of evaporative cooling, the performance of dry gels was consistently lower than that of hydrogels, underscoring the essential dual contribution of structural porosity and retained moisture in achieving optimal insulation. To investigate the relative contributions of thermal insulation and evaporation to the overall cooling performance, we analyzed the cooling effect of the LWM both in its fully hydrated state and after complete dehydration. Taking LWM80-8-30 as an example, under an 80 °C hot plate, the hydrated LWM80-8-30 exhibits a cooling capability of 51.1 °C, while the fully dried sample shows a cooling of 42.2 °C. The difference of 8.9 °C can thus be attributed to evaporative cooling, corresponding to 17.4% of the total cooling effect, whereas thermal insulation accounts for the remaining 82.6%. Using the same approach, the relative contributions of evaporation and insulation for other LWMs were calculated, as shown in Fig. S14.

### Spectral Performance and Outdoor Thermal Management Performance Testing of Ultra-Light Hydrogels

**Fig. 6:**
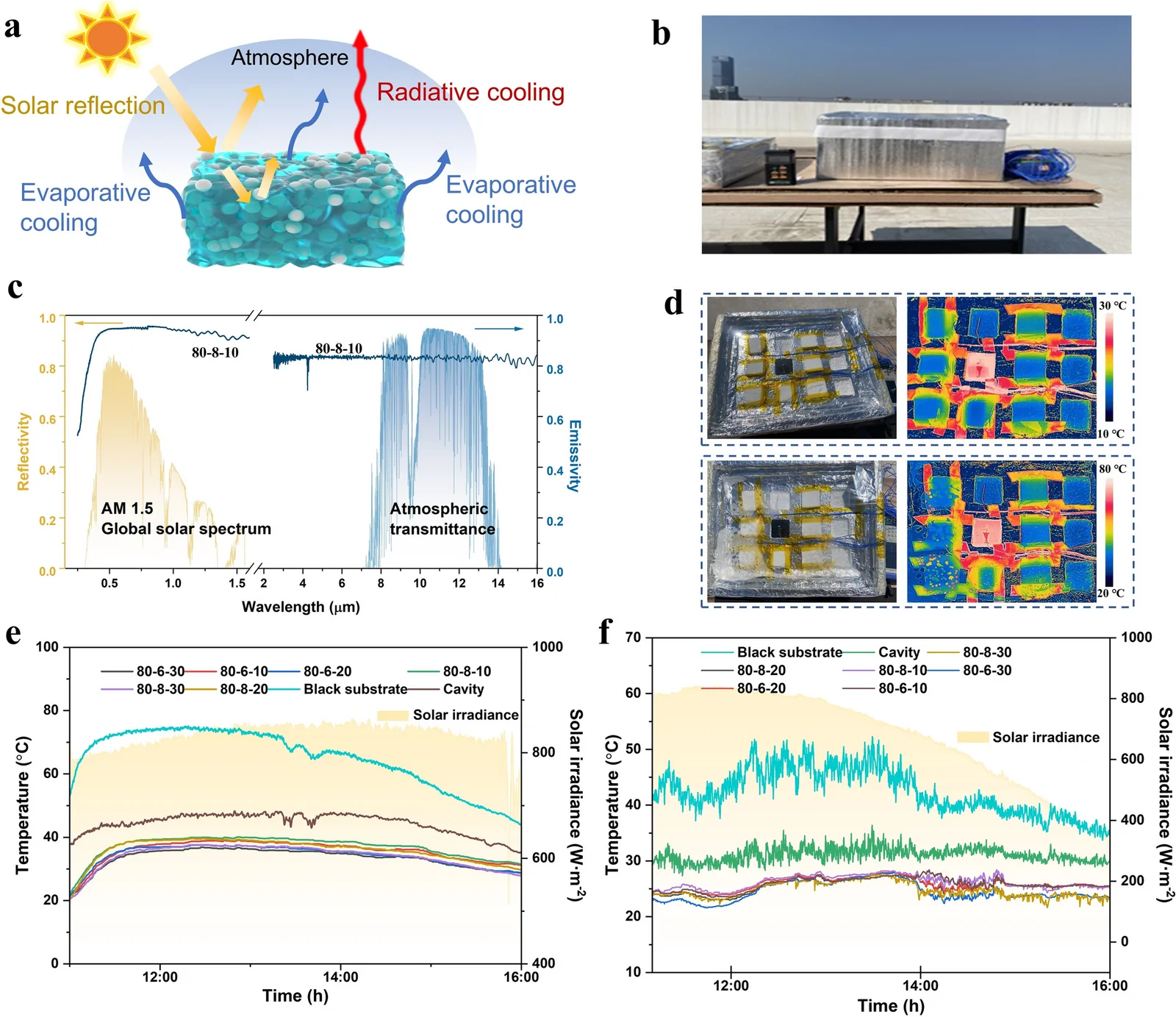
Spectral performance and outdoor thermal management performance testing of LWMs. **a** Schematic diagram of outdoor cooling mechanism, including solar reflection, thermal radiation, and evaporative cooling. **b** Actual image of the outdoor simulated test. **c** Optical performance of the LWM80-8-10 ultra-light hydrogel. The normalized ASTM G173 global solar spectrum and atmospheric transparency window are plotted as background. **d** Photograph image and infrared photograph of the outdoor simulated test with the device surface covered by a PE film. **e** LWMs temperature–time change curve of the outdoor simulated test with the device surface covered by PE film (March 17, 2025, Suzhou, China (31º15’N 120º43’E)). **f** LWMs temperature–time change curve of the outdoor simulated test with the device surface uncovered by PE film (March 24, 2025, Suzhou, China (31º15’N 120º43’E))

As illustrated by the cooling mechanism diagram in Fig. 6a, the presence of microspheres enables the hydrogel to efficiently reflect and diffusely scatter incident sunlight, resulting in a high solar reflectance [32, 33]. The hydrogel also exhibits a high infrared emittance (> 0.80) in the 2–16 μm range, primarily due to the intrinsic absorption features of water and the PNIPAM network. Water shows broad and intense mid-infrared absorption bands originating from O–H stretching and bending vibrations, strongly absorbing radiation throughout this region, while PNIPAM contributes additional absorption through the vibrational modes of its C=O, N–H, and C–H groups, particularly in the 6–12 μm range [52]. Together, these features give the hydrogel a consistently high emissivity across the mid-infrared spectrum. Figure 6b shows the specific outdoor testing scenario, and the optical properties of the ultra-light hydrogel with the lowest density (sample LWM80-8-10) are presented in Fig. 6c. The yellow-shaded region corresponds to the AM1.5 global solar spectrum, while the blue region represents the atmospheric transparency window. Based on this spectral distribution, the hydrogel exhibits an average solar reflectance of 0.94 in the 0.3–1.8 μm range, indicating that it can reflect the vast majority of incident sunlight and substantially reduce heat absorption. In addition, the material demonstrates an average emissivity of 0.84 in the mid-infrared region (8–13 μm), confirming its effective capability to dissipate absorbed heat as thermal radiation into the cold outer sky.

To further assess the outdoor thermal management performance of the LWMs, radiative cooling experiments were conducted under clear-sky conditions. Photographs of the testing setup and corresponding infrared images are shown in Fig. 6d. To minimize convective and conductive heat exchange between the chamber and the environment, the device was sealed with a transparent polyethylene (PE) film. Several small perforations were introduced in the film to allow water vapor diffusion from the hydrogel surface. However, in the PE-covered tests, the water vapor condensed on the film inevitably partially obstructed mid-infrared radiation within the atmospheric transparency window (Fig. 6d), thereby influencing the pure radiative cooling response. To decouple this effect, a second set of outdoor measurements was performed without sealing the surface, allowing the samples to be fully exposed to ambient air.

In Fig. 6d test conditions, the resulting temperature data for six LWMs were collected between 12:00 and 14:00 (including peak values), and the data are summarized in Fig. 6e. The measured temperatures for samples LWM80-8-30, LWM80-8-20, LWM80-8-10, LWM80-6-30, LWM80-6-20, and LWM80-6-10 were all close to 9 °C, corresponding to reductions of 9.0, 8.2, 7.3, 10.8, 10.0, and 8.5 °C relative to the cavity temperature. Therefore, both evaporative cooling and radiative heat loss synergistically contributed to the temperature reduction observed in the LWMs. Under test conditions without covering with PE film, the corresponding temperature–time curves are provided in Fig. 6f. Again, data from 12:00 to 14:00 were analyzed and averaged. Relative to the ambient cavity temperature, samples LWM80-8-30, LWM80-8-20, LWM80-8-10, LWM80-6-30, LWM80-6-20, and LWM80-6-10 achieved temperature drops of 5.5, 5.2, 4.5, 5.5, 4.9, and 5.2 °C, respectively. These findings demonstrate that even in a fully open environment with natural air convection, the combination of radiative and evaporative cooling remains highly effective, enabling the hydrogels to sustain substantial temperature reduction. For the present outdoor experiments, for relevant meteorological information on temperature, humidity, and wind speed, please refer to the supporting information Fig. S15, The temperature sensor was suspended centrally within the cavity volume, not in contact with any solid surface or the radiative sample, to measure the air temperature representative of the cavity interior; the uncertainty in the reported sub-ambient cooling values mainly arises from (a) the intrinsic accuracy of the temperature sensors and (b) short-term fluctuations in the ambient environment. The temperature sensors used in this study have an accuracy of ± 0.1 °C, which sets the lower bound of uncertainty in the cavity-ambient temperature difference. In addition, transient variations in ambient air temperature and wind speed introduce approximately ± 0.1–0.2 °C of additional variability, as determined from the standard deviation of the recorded environmental data. Overall, the sub-ambient cooling values in Fig. 6 are well above this combined uncertainty range, confirming that the observed cooling effect is statistically meaningful.

In addition, a quantitative comparison of the performance of LWMs with other hydrogels and aerogels is presented in Table 1 [31–33, 53, 55, 63–68]. The table compares key parameters, including density, water content, thermal conductivity, and cooling performance. The results indicate that LWMs with water contents ranging from 52.7% to 92.6% can achieve a density as low as 0.41 g cm^−3^, exhibit thermal conductivity lower than that of some aerogels, and demonstrate excellent cooling performance, with a temperature reduction of up to 10.8 °C. The data in Table 1 highlight that LWMs combine ultra-low density, superior thermal insulation, and strong cooling ability, outperforming many conventional hydrogels and even some aerogels.

**Table 1:** Quantitative comparison of LWMs with representative high-performance hydrogels and aerogels in terms of density, water content, thermal conductivity, and cooling performance

Classification	Density (g cm^−3^)	Water content (%)	Thermal conductivity (W m^−1^ K^−1^)	Cooling performance (°C)	References
Hydrogel	0.36	80	–	22.5	[32]
	0.95	96	–	–	[53]
	0.8–1	80	–	–	[55]
	1.59	61.1	–	17	[64]
	0.86	56.7	–	9	[65]
	–	20	0.484	12	[31]
Aerogel	–	–	0.026	10.2	[33]
	–	–	0.035	7.5	[66]
	–	–	0.056	–	[67]
	0.36	–	0.062	–	[68]
	0.15–0.3	–	0.039	9.1	[63]
LWM	0.041	52.7 ~ 92.6	< 0.04	10.8	This work

## Conclusions

In summary, this work successfully designed and synthesized an ultra-lightweight PNIPAM-based hydrogel reinforced with hollow foaming microspheres, thereby realizing the concept of LWM. By systematically optimizing synthesis parameters, this work achieved a record-low density of 0.041 g cm^−3^ while preserving a high water content of 52.7 wt%, and the density is adjustable between 0.041 and 0.532 g cm^−3^, overcoming the long-standing trade-off between lightweight structure and water retention in hydrogel systems. Importantly, the incorporation of hollow microspheres not only reduced density but also enhanced mechanical robustness, allowing the hydrogel to maintain structural stability under deformation and thermal conditions. Thermal management studies further revealed the multifunctionality of this material. The hydrogel exhibited ultra-low thermal conductivity in the range of 0.034–0.039 W m^−1^ K^−1^, resulting in a substantial equilibrium temperature difference of 47.6–51.4 °C in hot-stage insulation tests, highlighting its insulation performance. In addition, its outstanding optical performance, characterized by a high solar reflectance (0.94) and infrared emittance (0.84), enabled efficient radiative dissipation of heat. These spectral properties, combined with moisture-assisted evaporative cooling, produced remarkable sub-ambient temperature reductions of up to 10.8 °C in outdoor experiments under direct sunlight. Collectively, these results establish the PNIPAM-based LWM as a distinctive class of ultra-light, multifunctional, water-based material that integrates low density, enhanced mechanical strength, superior thermal insulation, and highly efficient passive radiative cooling. The development of such “light water” hydrogels establishes a foundational material platform whose lightweight architecture and thermal functionality are validated through representative scenarios such as radiative cooling and thermal insulation, while also offering promising pathways for future extensions.

## Supplementary Information

Below is the link to the electronic supplementary material.

Supplementary file1 (DOCX 12507 kb)
