# Supplementary material for: Ultra-Light Poly(N-isopropylacrylamide) Hydrogels: Light Weight Water Materials for Passive Thermal Management via Insulation and Cooling
Source: Nanomicro Lett. 2026 Jan 28;18:225. doi: 10.1007/s40820-025-02057-9 (PMC12847538; doi:10.1007/s40820-025-02057-9)
Supplement: Supplementary file 1 — Supplementary file1 (DOCX 12507 kb) [file 40820_2025_2057_MOESM1_ESM.docx]

Supporting Information for

**Ultra-Light** **Poly(N-isopropylacrylamide) Hydrogels-Lightweight Water Materials for Passive Thermal Management via Insulation and Cooling**

Xueyan Hu^1,2,^^†^, Siyuan Dou^1,†^, Yiming Liu^1,2^, Yaru Li^1,2^, Caixia Yu^1^, Jin Wang^1,2,*^

^1^ Key Laboratory of Multifunctional Nanomaterials and Smart Systems, Suzhou Institute of Nano-Tech and Nano-Bionics, Chinese Academy of Sciences, Suzhou 215123, P. R. China.

^2^ School of Nano-Tech and Nano-Bionics, University of Science and Technology of China, Hefei 230026, P. R. China

^†^Xueyan Hu and Siyuan Dou contributed equally to this work.

*Corresponding author. E-mail: [jwang2014@sinano.ac.cn](mailto:jwang2014@sinano.ac.cn) (Jin Wang)

**Supplementary Figures**


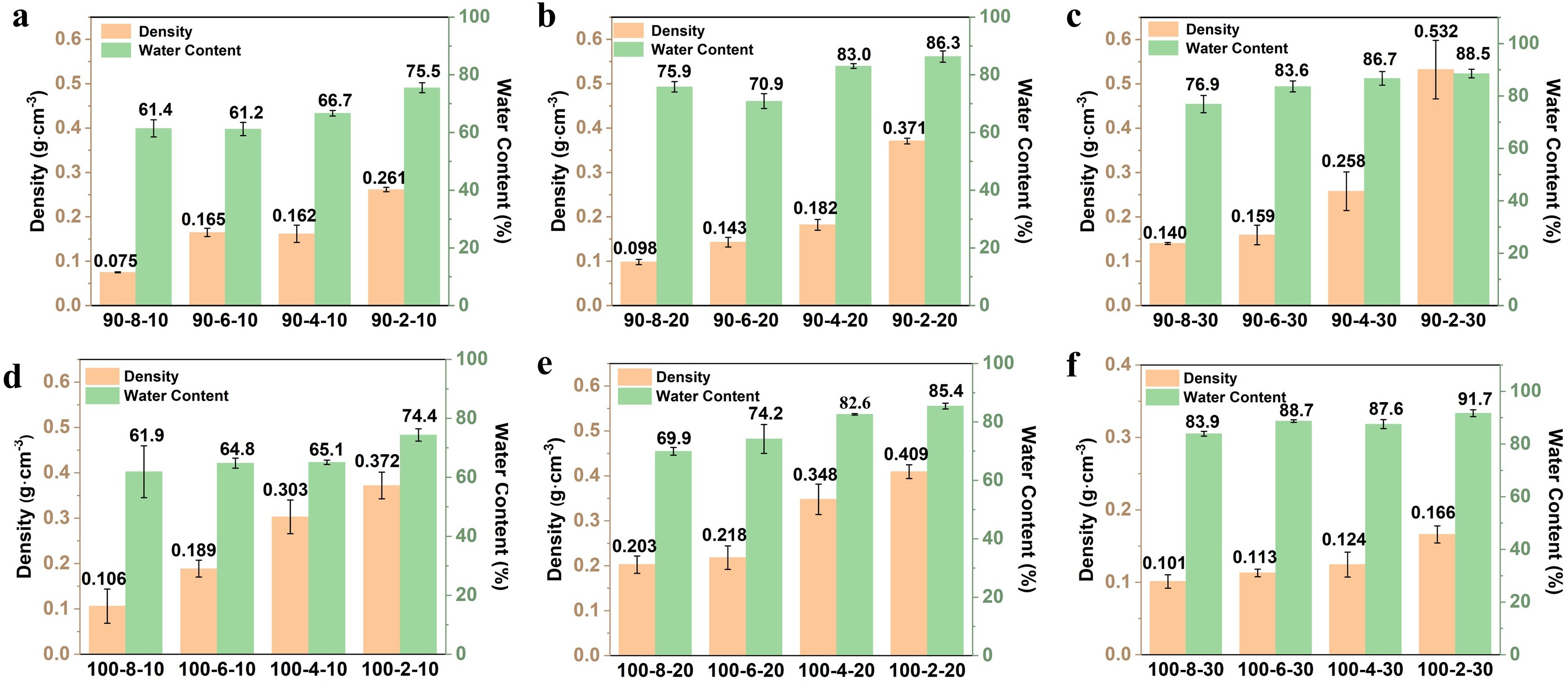


**Fig. S1** **a–b** Density and water content bar charts of the ultra-light hydrogels with crosslinking temperatures of 90 °C and deionised water additions of 10 mL, 20 mL, and 30 mL, respectively. **d–f** Column charts showing the density and water content of LWMs with 10 mL, 20 mL, and 30 mL of deionised water added at a cross-linking temperature of 100 °C. (The error bars represent the standard deviation obtained from three measurements.)


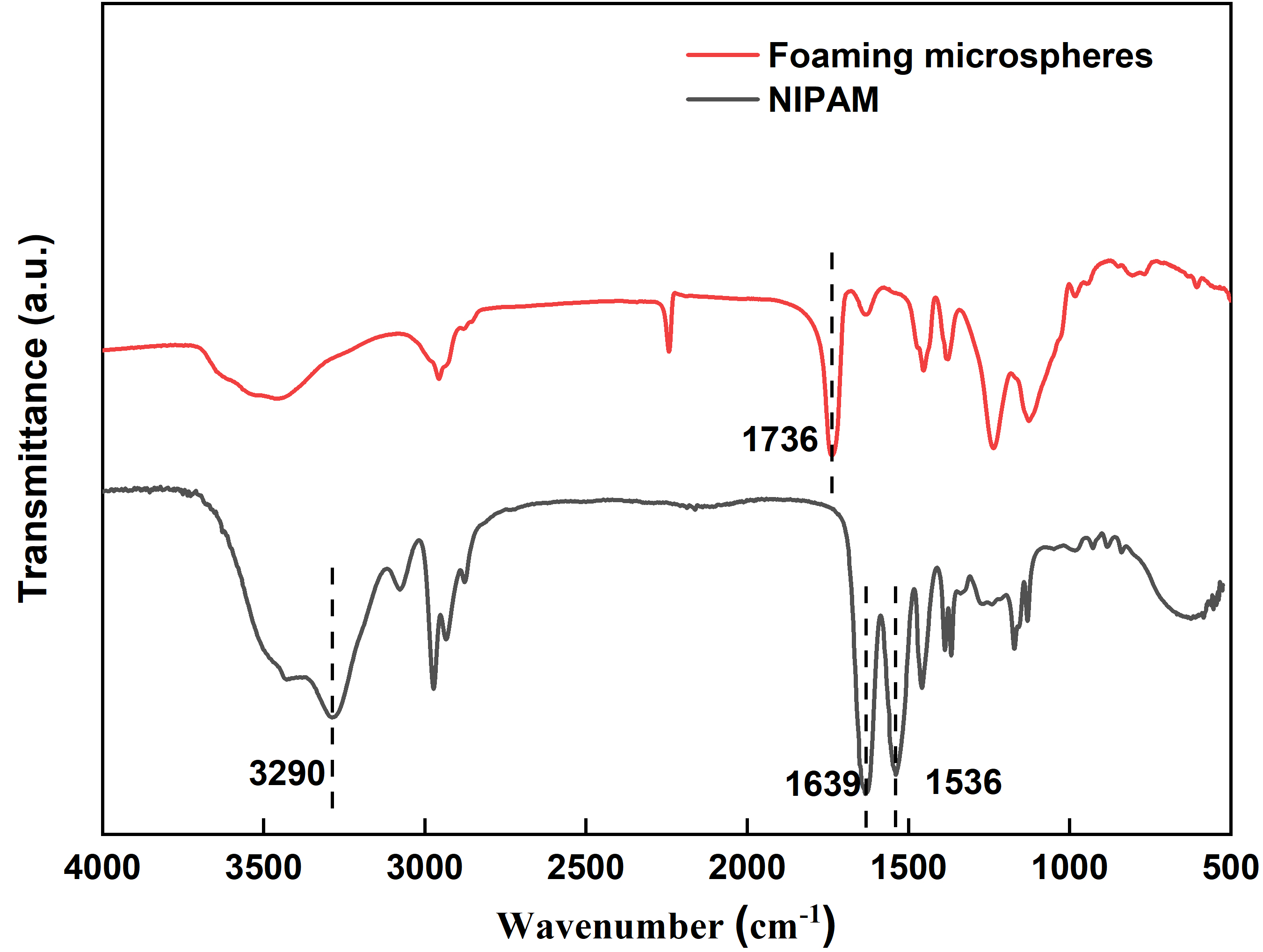


**Fig.** **S2** FT-IR spectroscopy of foaming microsphere and NIPAM


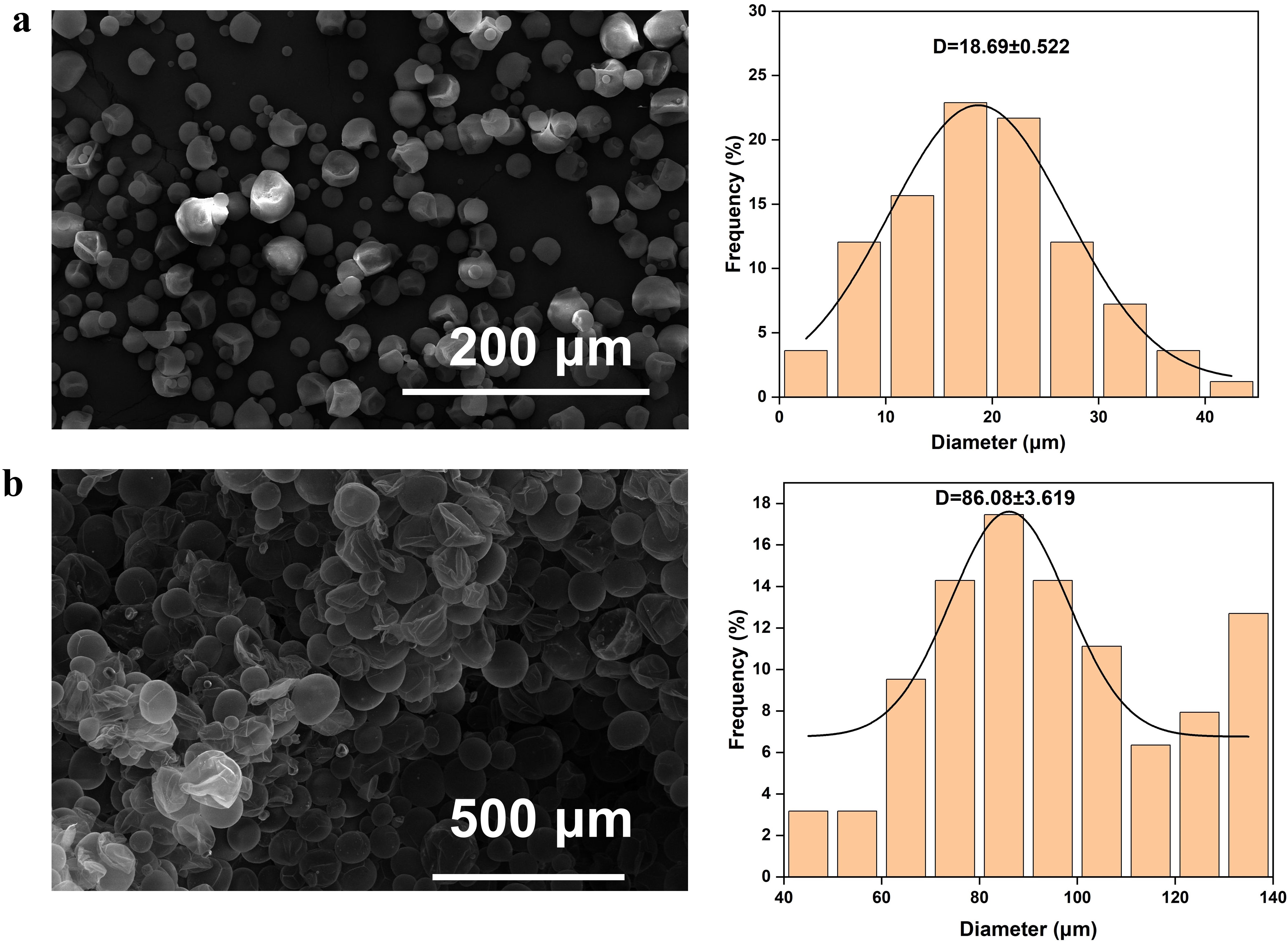


**Fig.** **S3** Scanning electron microscope images of pre-reaction foaming microspheres (**a**) and post-reaction foaming microspheres (**b**), and corresponding pore size distribution diagrams


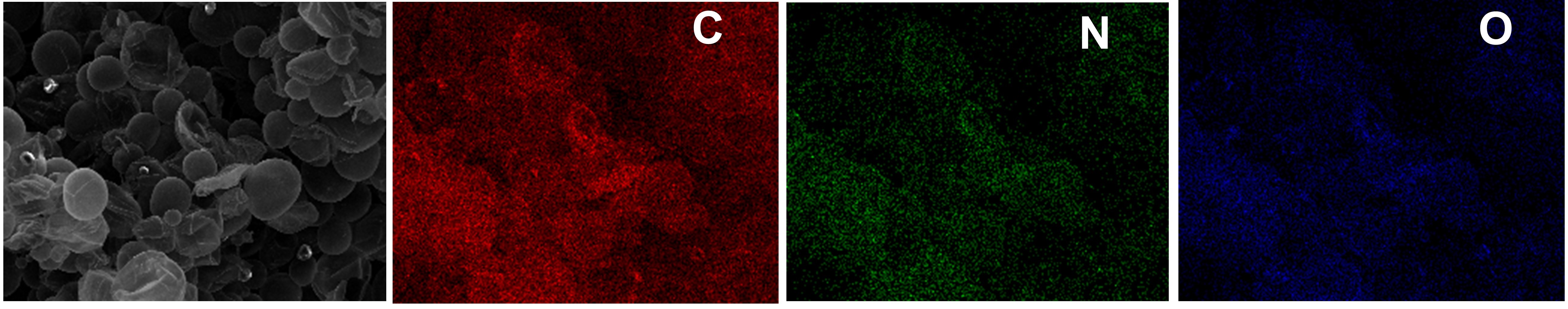


**Fig.** **S4** EDS mapping image corresponding to the SEM image of post-reaction foaming microspheres


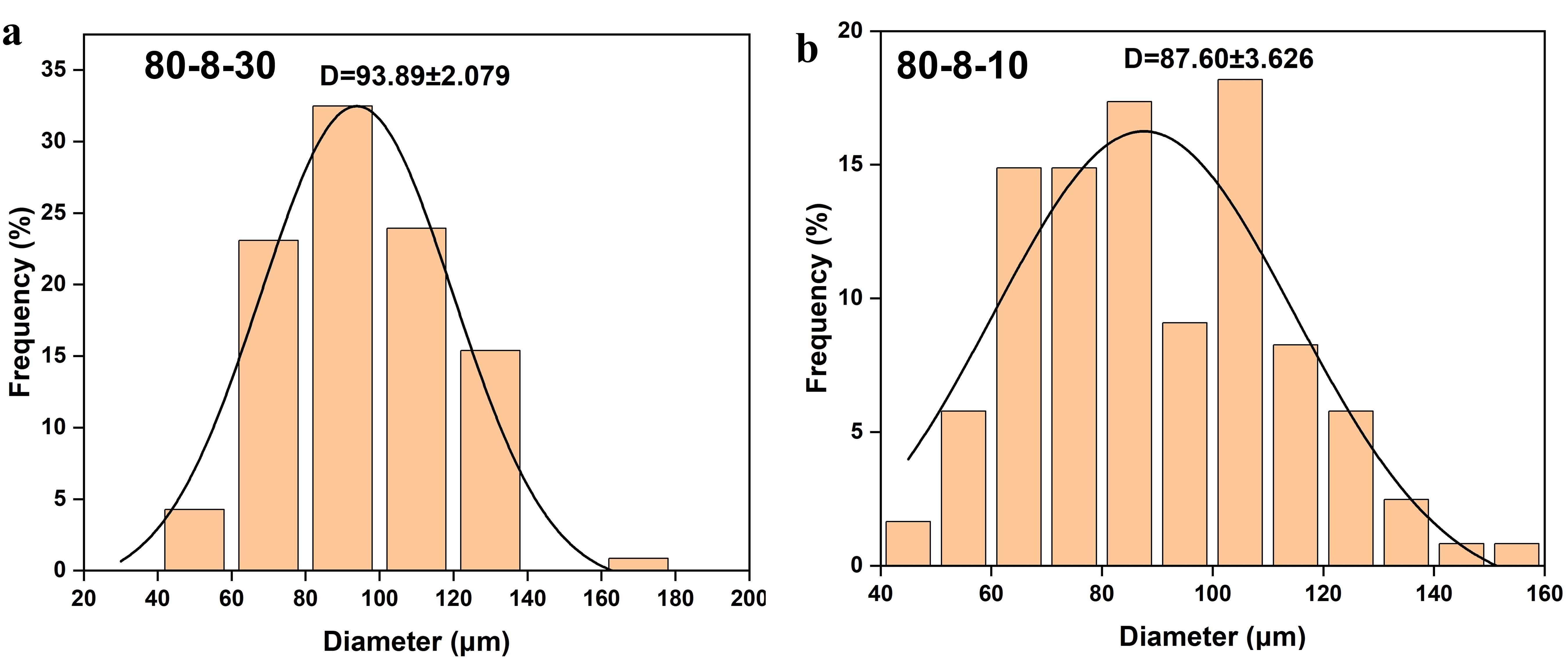


**Fig.** **S5** Pore size distribution diagrams of dry gels LWM80-8-30 and LWM80-8-10


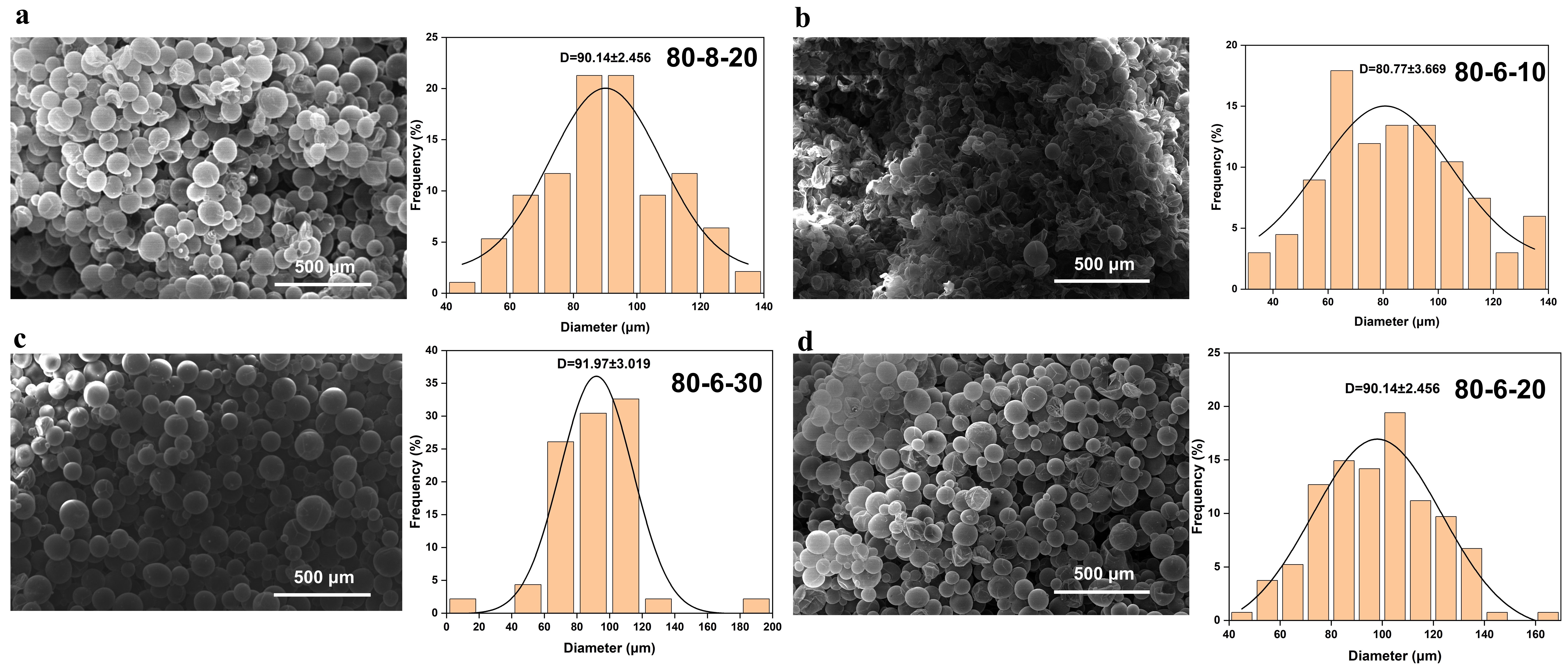


**Fig.** **S6** Scanning electron microscope images of dry gels LWM80-8-20, LWM80-6-10, LWM80-6-30, and LWM80-6-20 and corresponding pore size distribution diagrams


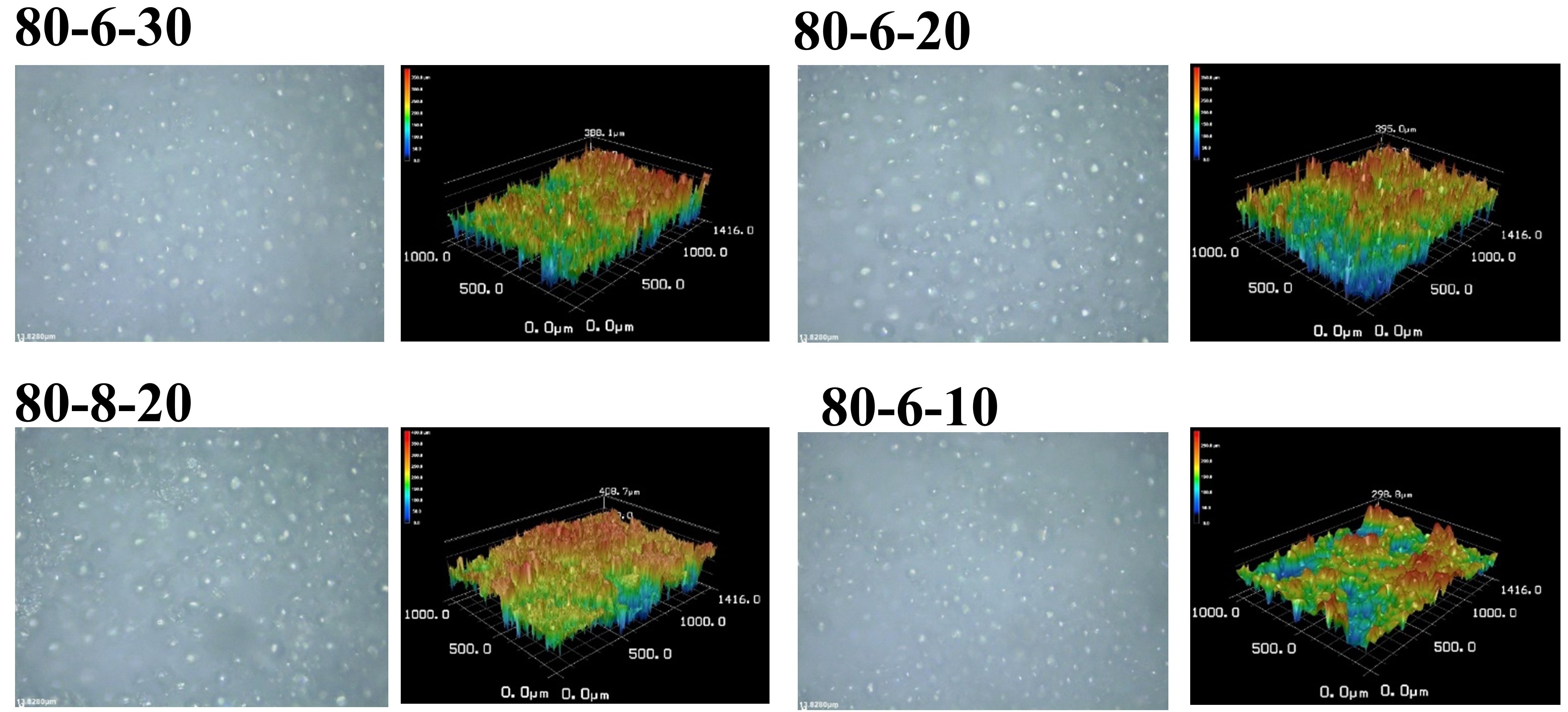


**Fig.** **S7** Three-dimensional surface morphology of hydrogels LWM80-6-30, LWM80-6-20, LWM80-8-20, and LWM80-6-10


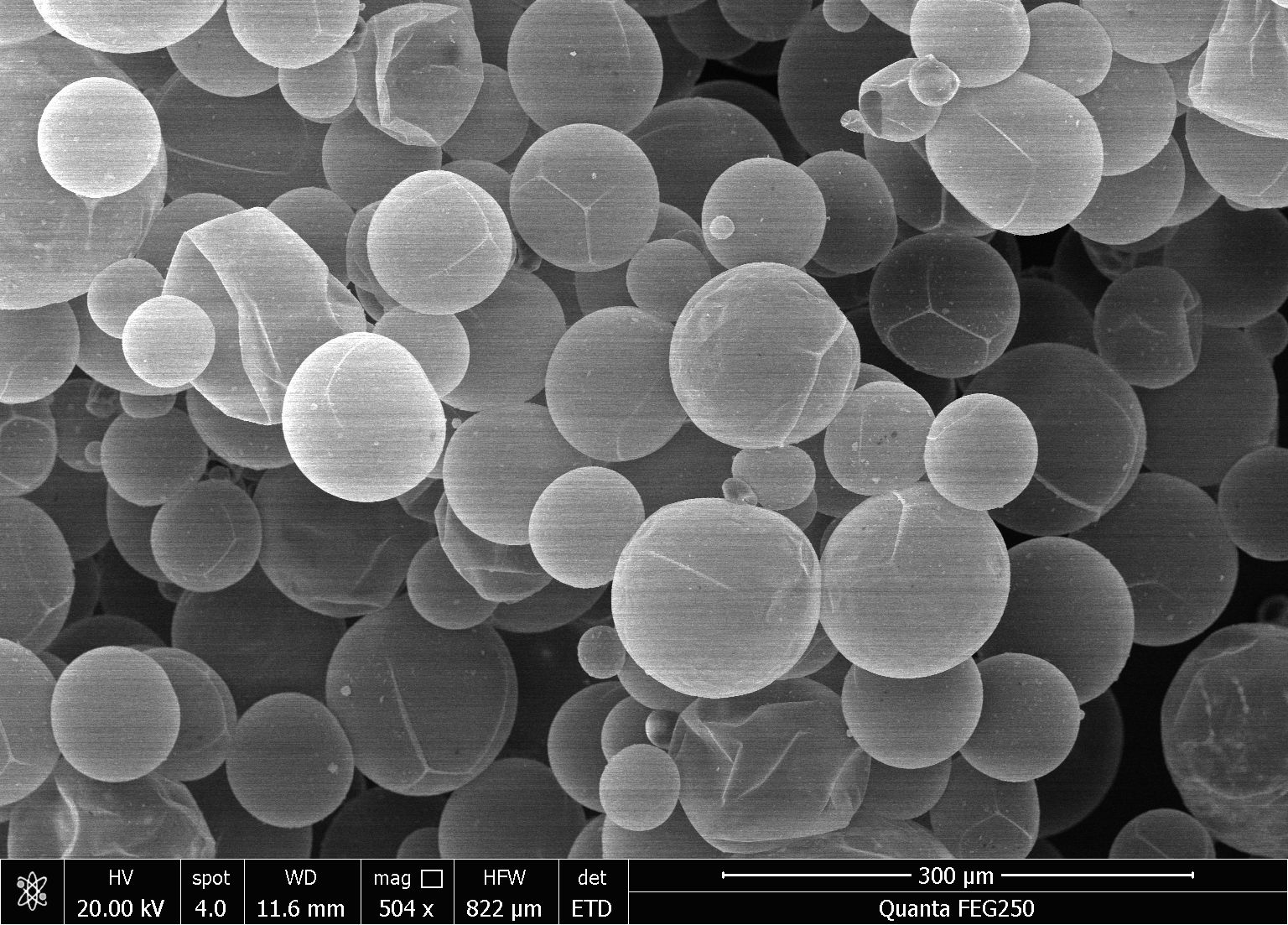


**Fig.** **S8** SEM image of LWM80-8-10 after three thermal cycles (30 min at ambient temperature and 30 min on an 80 °C hot plate; one cycle lasts 1 h)


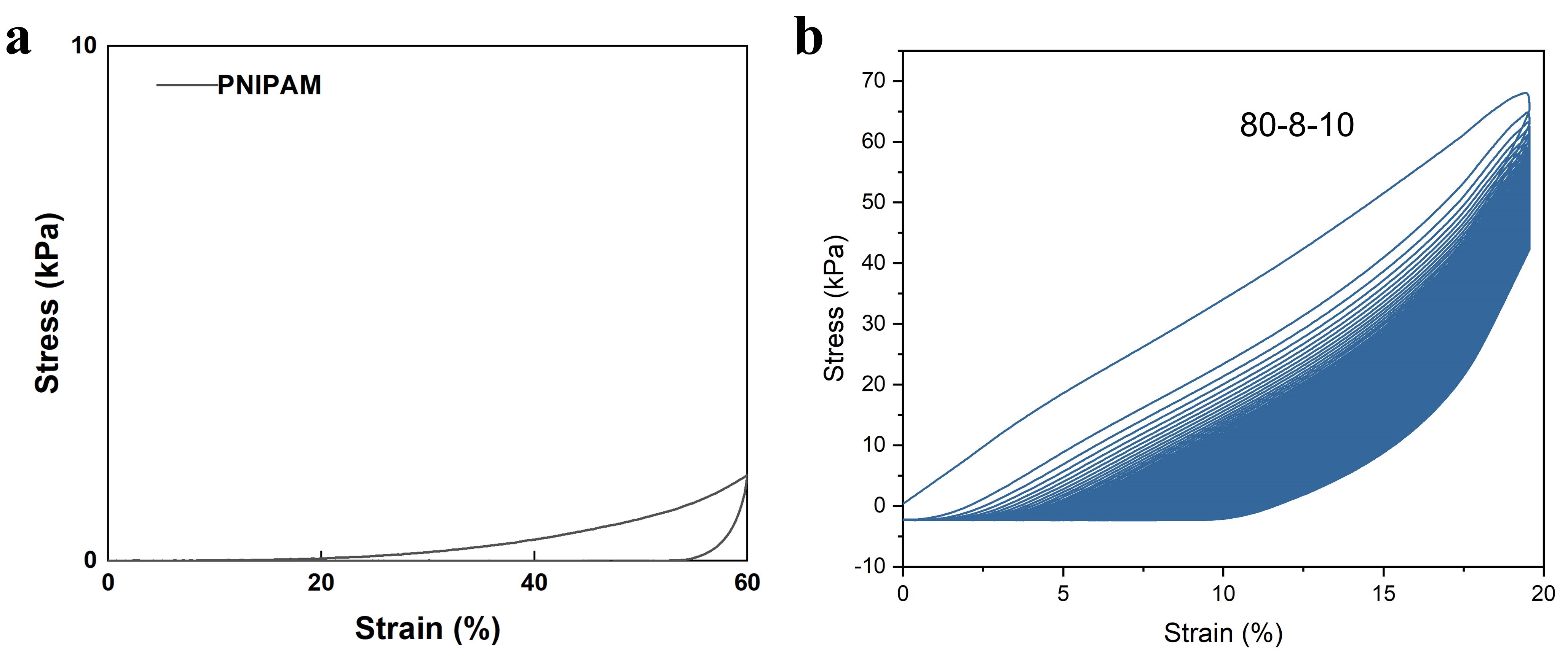


**Fig.** **S9** Stress-strain curve of a loading-unloading cycle of a pure PNIPAM hydrogel


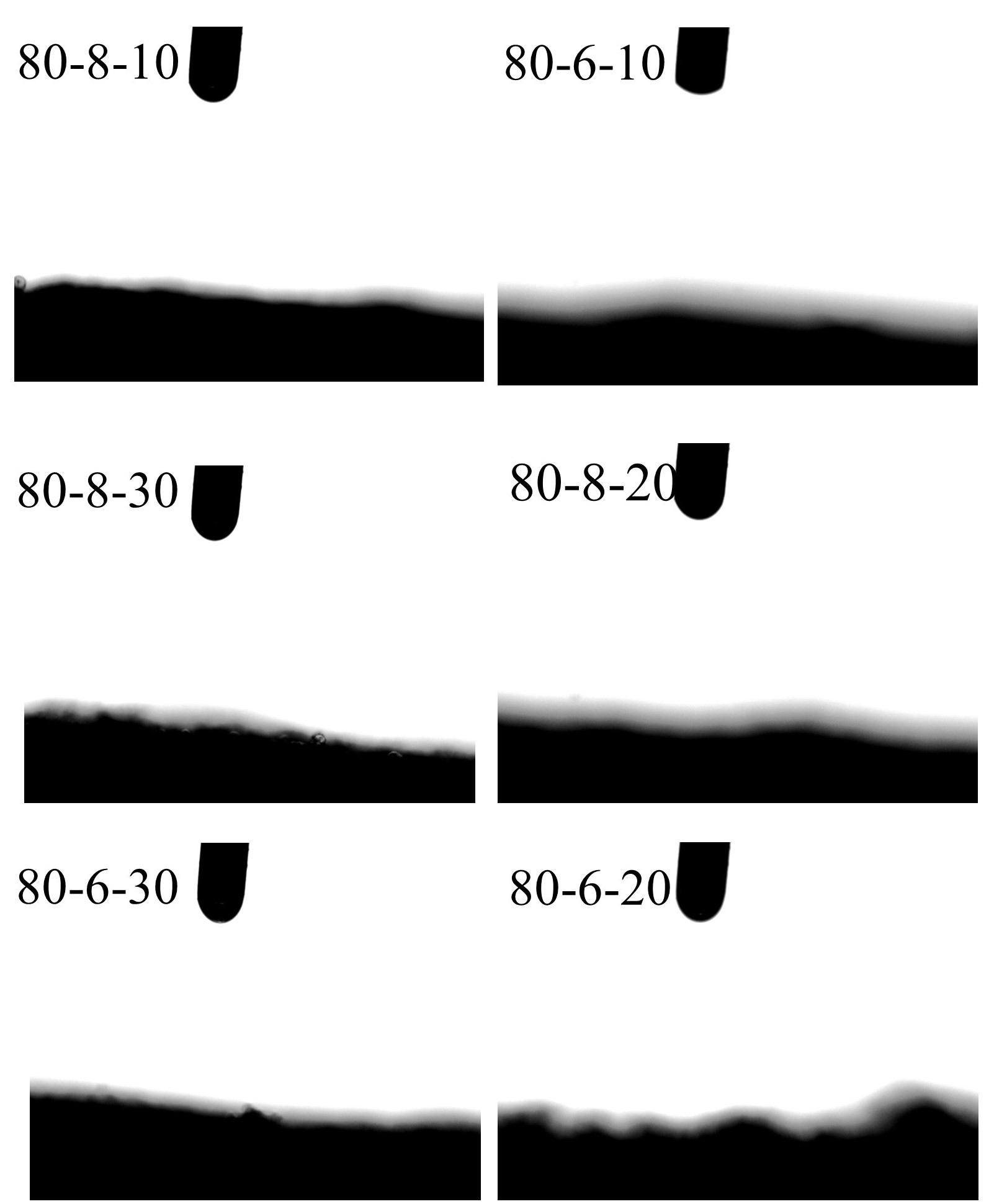


**Fig.** **S10** Contact angle of LWM80-8-10, LWM80-6-10, LWM80-8-30, LWM80-8-20, LWM80-6-30, and LWM80-6-20 hydrogels


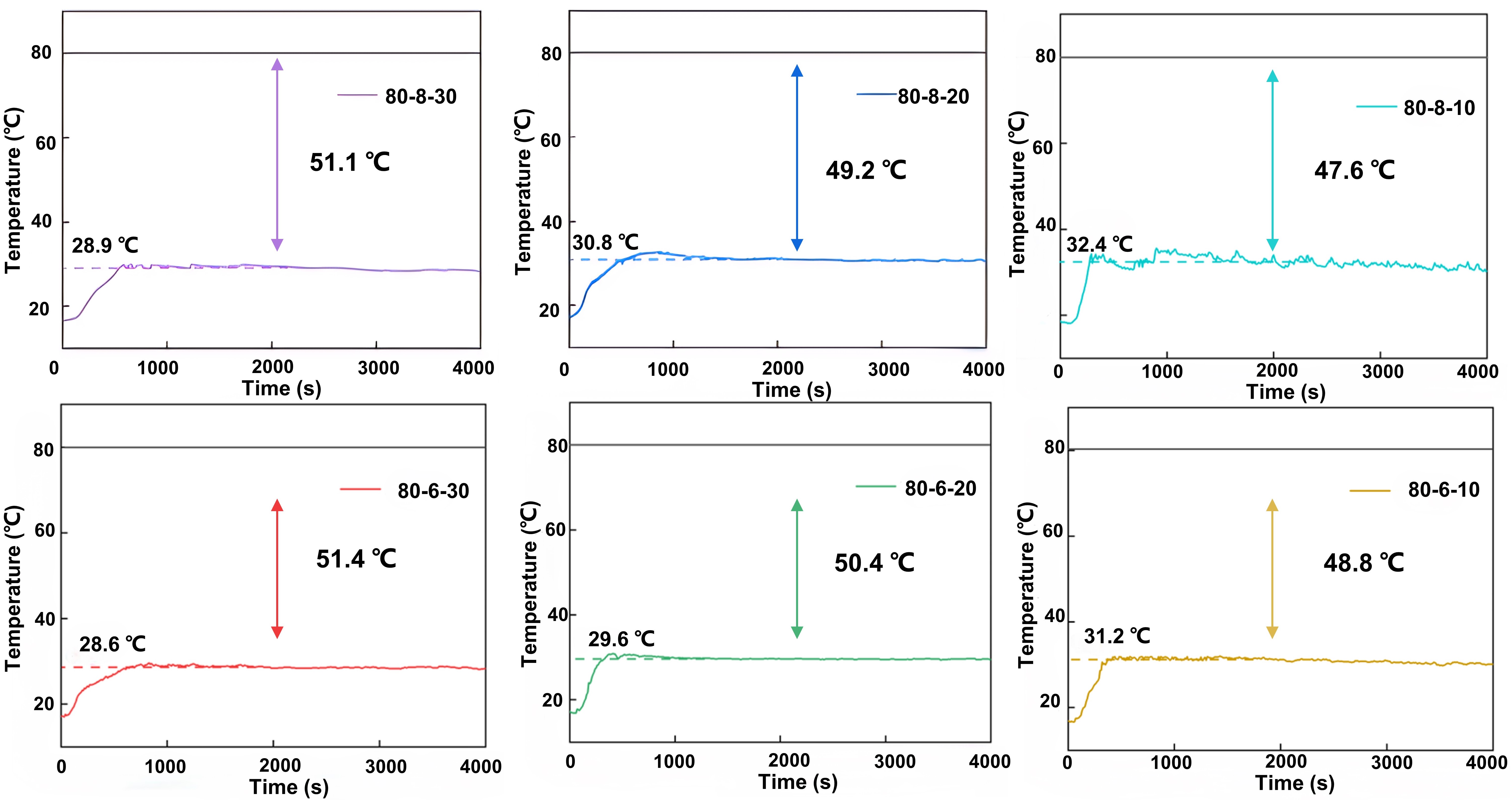


**Fig.** **S11** Temperature-time change curves of hydrogel hot plate experiments at LWM80-8-30, LWM80-8-20, LWM80-8-10, LWM80-6-30, LWM80-6-20, and LWM80-6-10


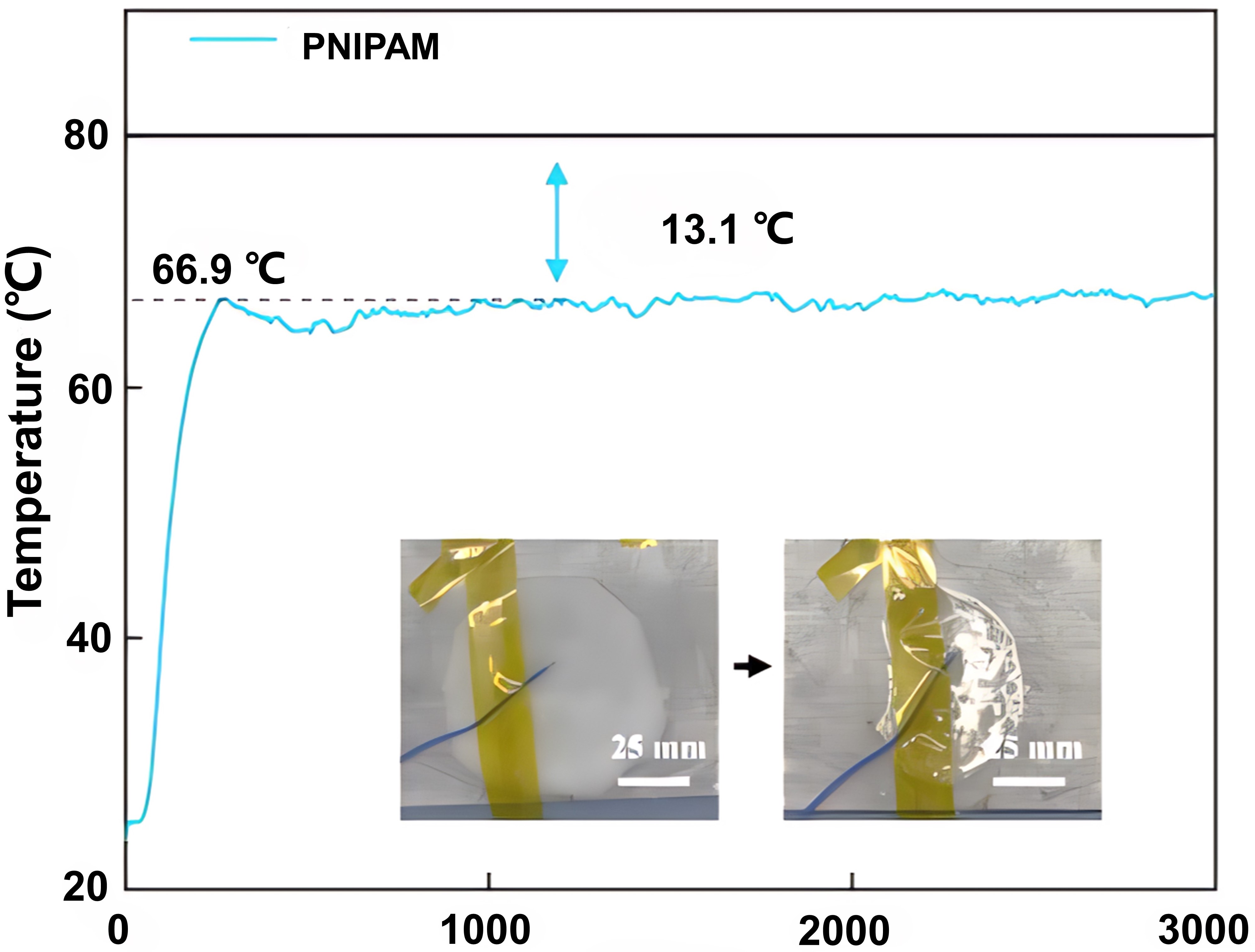


**Fig.** **S12** Temperature-time change curve of PNIPAM hydrogel hot plate experiment


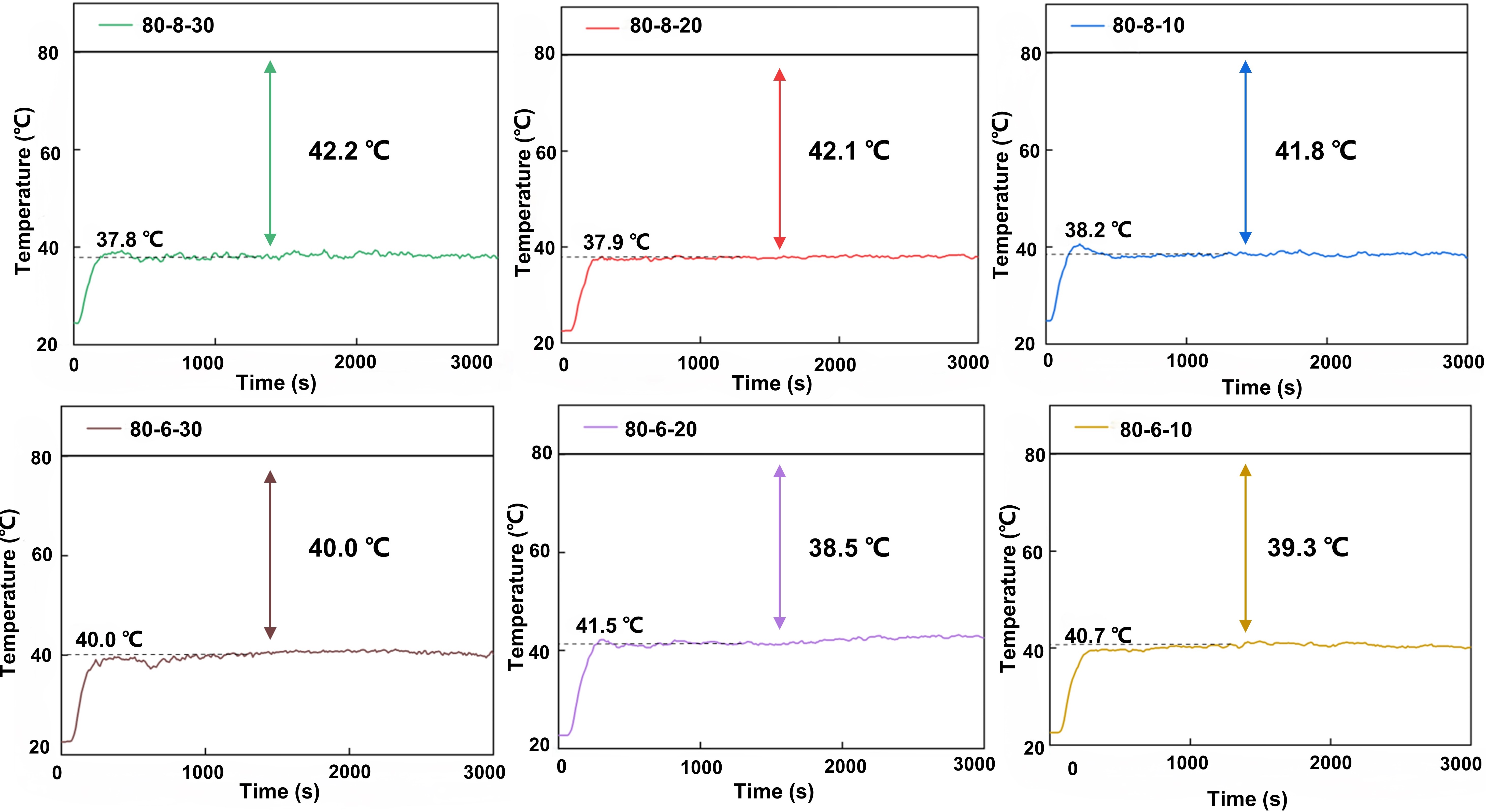


**Fig.** **S13** Temperature-time change curves for dry gel hot plate experiments at LWM80-8-30, LWM80-8-20, LWM80-8-10, LWM80-6-30, LWM80-6-20, and LWM80-6-10


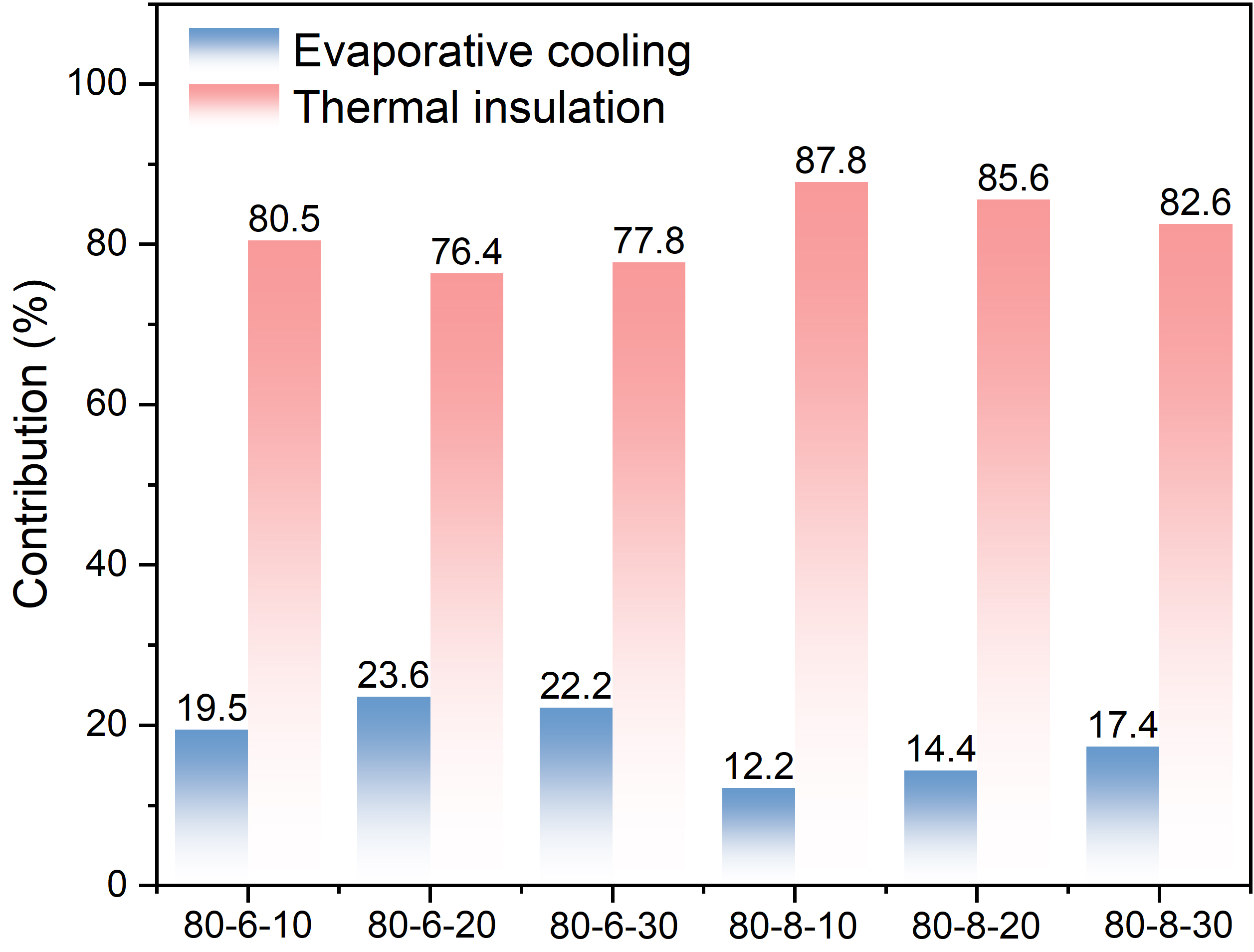


**Fig.** **S14** Percentage of water evaporation and thermal insulation of LWM hydrogels


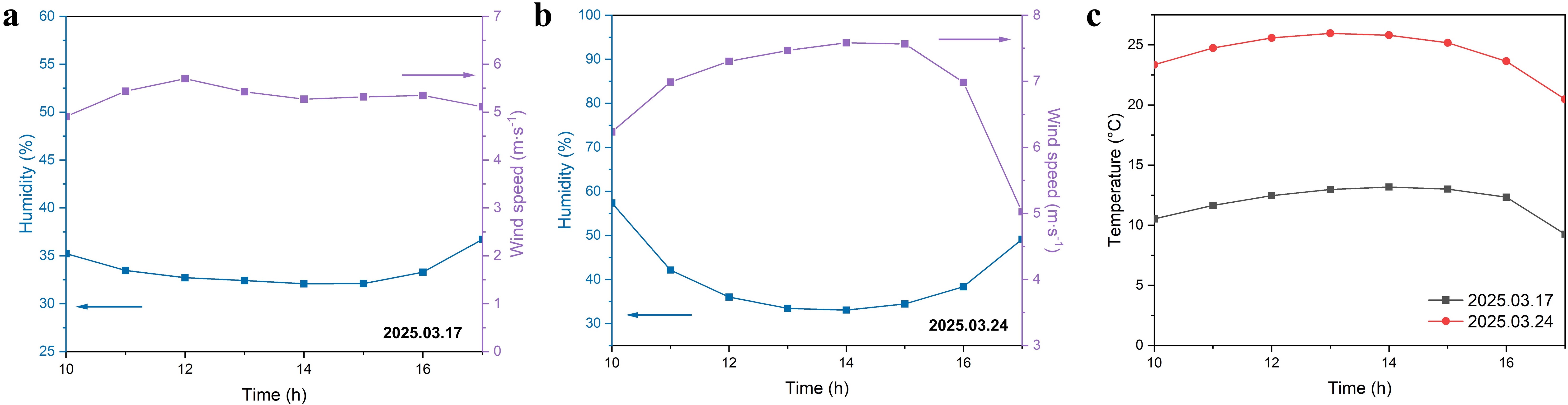


**Fig.** **S15** Humidity, wind speed and meteorological temperature during outdoor testing
